# The differential expression of alternatively polyadenylated transcripts is a common stress-induced response mechanism that modulates mammalian mRNA expression in a quantitative and qualitative fashion

**DOI:** 10.1261/rna.055657.115

**Published:** 2016-09

**Authors:** Ina Hollerer, Tomaz Curk, Bettina Haase, Vladimir Benes, Christian Hauer, Gabriele Neu-Yilik, Madhuri Bhuvanagiri, Matthias W. Hentze, Andreas E. Kulozik

**Affiliations:** 1 Molecular Medicine Partnership Unit (MMPU), Heidelberg 69120, Germany; 2 European Molecular Biology Laboratory (EMBL), Heidelberg 69117, Germany; 3 Department of Pediatric Oncology, Hematology and Immunology, University of Heidelberg, Heidelberg 69120, Germany; 4 Faculty of Computer and Information Science, University of Ljubljana, Ljubljana 1001, Slovenia

**Keywords:** alternative polyadenylation, 3′ end processing, stress, mRNA-seq, polyadenylation site mapping

## Abstract

Stress adaptation plays a pivotal role in biological processes and requires tight regulation of gene expression. In this study, we explored the effect of cellular stress on mRNA polyadenylation and investigated the implications of regulated polyadenylation site usage on mammalian gene expression. High-confidence polyadenylation site mapping combined with global pre-mRNA and mRNA expression profiling revealed that stress induces an accumulation of genes with differentially expressed polyadenylated mRNA isoforms in human cells. Specifically, stress provokes a global trend in polyadenylation site usage toward decreased utilization of promoter-proximal poly(A) sites in introns or ORFs and increased utilization of promoter-distal polyadenylation sites in intergenic regions. This extensively affects gene expression beyond regulating mRNA abundance by changing mRNA length and by altering the configuration of open reading frames. Our study highlights the impact of post-transcriptional mechanisms on stress-dependent gene regulation and reveals the differential expression of alternatively polyadenylated transcripts as a common stress-induced mechanism in mammalian cells.

## INTRODUCTION

Cells adapt to a multitude of potentially harmful stimuli by adjusting the expression of genes important for their response to stress or inflammation. In this context, post-transcriptional mechanisms play a major role in regulating gene expression by fine-tuning RNA and protein levels and by augmenting or buffering transcriptional effects (Komili and Silver 2008).

Regulated 3′ end processing has recently emerged as an important mechanism to control gene expression. It can do so in a quantitative manner by stimulating or inhibiting mRNA processing at specific polyadenylation [poly(A)] sites and thus affect mRNA and protein abundances (Nazeer et al. 2011; Di Giammartino et al. 2013). This is highlighted by the expression of the prothrombin (*F2*) gene that is regulated via stimulated 3′ end processing in cells exposed to stress and inflammatory stimuli (Danckwardt et al. 2011). Further, alternative polyadenylation (APA) can qualitatively affect the expression of genes harboring more than one functional poly(A) site and may trigger the expression of distinct mRNA and protein isoforms (Elkon et al. 2013; Tian and Manley 2013). Both mechanisms contribute to important decisions in development and differentiation, or, if deregulated, can cause disease, including cancer (Danckwardt et al. 2008; Hollerer et al. 2014).

Alternative polyadenylation may alter the expression of mRNA and protein isoforms when occurring within a gene's intronic or coding region, a mechanism referred to as coding-region (CR-) APA (Di Giammartino et al. 2011). APA affecting 3′UTR length, called 3′UTR-APA, can lead to global shortening of 3′UTRs in cancer and in proliferating cells (Sandberg et al. 2008; Mayr and Bartel 2009; Elkon et al. 2012; Lin et al. 2012; Bava et al. 2013) or can provoke transcriptome-wide 3′UTR lengthening in differentiation and development (Shepard et al. 2011; Hoque et al. 2013). A recent study reported cold shock conditions to globally influence 3′UTR lengths, which regulates gene expression according to the circadian clock (Liu et al. 2013). In addition, APA occurs upon UV-induced damage in yeast (Graber et al. 2013; Yu and Volkert 2013) and can stress-dependently influence the expression of specific genes, such as *HSP70.3*, under heat shock conditions (Kraynik et al. 2015). Alternative polyadenylation may thus also globally influence mammalian gene expression in conditions of cellular stress. Early work on alternative polyadenylation in proliferating and cancer cells suggested that APA-provoked changes in 3′UTR length inversely correlated with the expression levels of the respective mRNAs and proteins, likely caused by modified miRNA and RBP binding (Sandberg et al. 2008; Mayr and Bartel 2009). This hypothesis was challenged by other studies that reported 3′UTR length to have a limited effect on mRNA and protein expression levels in yeast and mice (Spies et al. 2013; Gruber et al. 2014; Gupta et al. 2014). As a most interesting new dimension, a recent report implicated 3′UTR-APA to influence the localization of newly translated proteins and thus described an essential cellular role of APA beyond modulating mRNA abundance (Berkovits and Mayr 2015).

We have here implemented a strategy to define the global role of alternative polyadenylation in the mammalian cellular stress response and performed high-resolution poly(A) site mapping combined with mRNA- and pre-mRNA-seq on stressed and unstressed cells to unravel the impact of APA on stress-induced changes in gene expression on a global scale. These data enabled us to obtain an unbiased view on how cellular stress affects poly(A) site choice and how this mechanism contributes to stress-regulated gene expression.

## RESULTS

### Stress induces the expression of a large number of alternatively polyadenylated transcripts and differentially regulates promoter-distal and promoter-proximal poly(A) sites

Previous results of our group revealed stimulated 3′ end processing to increase the expression of the prothrombin (*F2*) gene in cells treated with anisomycin (Danckwardt et al. 2011), a compound that induces ribotoxic stress by inhibiting the peptidyl transferase activity of the ribosome, and thereby stimulates stress-activated protein kinases. We now set out to explore whether stress treatment also influences poly(A) site choice in mammalian cells and, if so, to what extent stress-provoked APA contributes to transcriptome-wide changes in gene expression levels.

We globally mapped functional poly(A) sites in HEK T293 cells treated with anisomycin or the solvent control DMSO performing the 3′T-fill method (Wilkening et al. 2013) on three independent biological replicates and analyzed the differential use of these poly(A) sites under these conditions. For creating 3′T-fill libraries, we fragmented and reverse transcribed cytoplasmic RNA with an oligo(dT) primer establishing cDNA libraries derived from polyadenylated RNA fragments only. In the cluster station of the sequencer, poly(A) tails were first saturated with nonlabeled dTTP residues to prevent the subsequent sequencing reaction from starting within the poly(A) tail. The sequencing reaction then started right before the poly(A) tail with the first incorporated labeled dinucleotide reflecting the site of mRNA polyadenylation.

**FIGURE 1.**
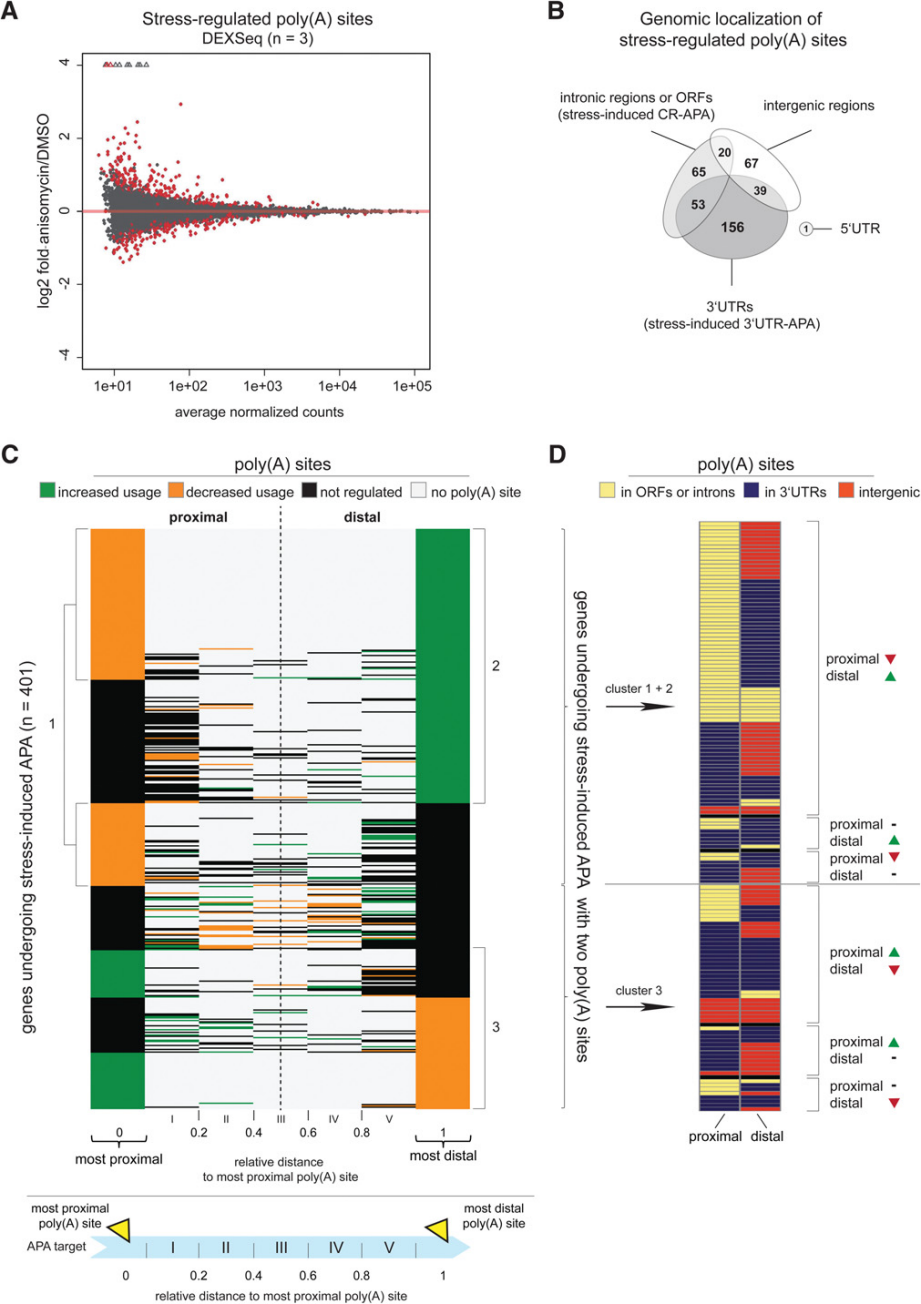
Stress induces alternative polyadenylation in a large number of RNAs and globally leads to the increased usage of distal and the decreased usage of proximal poly(A) sites. (*A*) DEXSeq analysis displaying stress-regulated poly(A) sites as detected in 3′T-fill [significantly regulated poly(A) sites are represented as red dots; *P*_adj_ < 0.1]. 3′T-fill analyses were performed on three independent biological replicates. (*B*) Venn diagram displaying genes with at least one stress-regulated poly(A) site and the distribution of these sites across different genomic regions, including intronic regions or open reading frames (ORFs) and 3′UTRs, stimulating coding-region (CR-) and 3′UTR-APA, respectively, as well as 5′UTRs and intergenic regions. (*C*) The gene region between the most proximal and the most distal poly(A) site was separated into five equal sections I–V and scanned for poly(A) sites (see cartoon *beneath*). In this way, poly(A) sites lying between the most proximal and the most distal poly(A) site could thus be classified as “proximal” or “distal” depending on their relative distance to the most proximal poly(A) site. The heat map displays the stress-dependent regulation of the utilization of all poly(A) sites on an individual gene basis. Poly(A) sites showing a stress-induced increased usage are shown in green, poly(A) sites with decreased usage are depicted in orange, and poly(A) sites that were not stress-dependently regulated are displayed in black. The data were ordered according to the regulation of the most distal poly(A) site. APA targets were enriched for genes with down-regulated usage of their most proximal poly(A) site (cluster 1) and increased usage of their most distal poly(A) site (cluster 2). Genes that showed the opposite regulation of poly(A) site usage with increased utilization of the proximal and decreased utilization of the most distal poly(A) site were grouped into cluster 3. (*D*) The regulated poly(A) sites of the cluster 1 and 2 genes, which showed decreased proximal and/or increased distal poly(A) site usage, as well as for the cluster 3 genes, which showed the opposite trend, were categorized according to the genomic region they occurred in. For simplicity, only genes with two poly(A) sites were considered in this analysis. Poly(A) sites lying in introns or ORFs are shown in yellow, those lying in 3′UTRs are depicted in dark blue, and those occurring in intergenic regions are shown in dark orange.

Originally developed in yeast, 3′T-fill yielded highly reproducible data in human cells in our hands. In total, we detected 23,878 high-confidence poly(A) sites supported by >10 reads per sample, including 1218 (5%) previously unannotated sites (Derti et al. 2012) within 12,085 transcripts (Supplemental Fig. S1A). We applied DEXSeq to our 3′T-fill data set to test for differential usage of the poly(A) sites in each gene (Anders et al. 2012). We counted the number of 3′T-fill reads at each poly(A) site of a gene in stress and control samples and estimated the stress-induced change of the “expression” of each poly(A) site relative to the “expression” of other poly(A) sites of the same gene. Using this approach, we found 401 genes that showed a differential expression of their poly(A)^+^ mRNA isoforms upon stress treatment at a false discovery rate (FDR) of 10% (DEX-Seq *P*_adj_ < 0.1) (Fig. 1A; Supplemental Table S1), revealing that cellular stress provokes alternative polyadenylation. A DAVID analysis (Dennis et al. 2003) showed that these APA targets were significantly enriched for genes encoding nuclear proteins but exerts diverse biological functions and do not cluster in any defined cellular mechanisms or biological pathways (Supplemental Fig. S1B).

We next defined stress-provoked CR- and 3′UTR-APA events by identifying the genomic regions containing stress-regulated poly(A) sites. We therefore analyzed all transcript isoforms of the individual genes undergoing stress-induced APA that were annotated for the human reference genome GRCh37 as provided by Ensembl 73. First, we defined regulated poly(A) sites occurring in the 3′UTR of any annotated gene transcript as 3′UTR-APA sites and identified 248 targets in which stress-dependent 3′UTR-APA altered 3′UTR length. We subsequently defined the remaining poly(A) sites as CR-APA events when lying in upstream intronic regions or within open reading frames of annotated transcripts, which yielded 138 genes whose mRNAs were influenced by CR-APA. We then assigned each intergenic poly(A) site to the next upstream lying annotated gene irrespective of its distance from the gene's 3′ end, being aware of the fact that those poly(A) sites could, in principle, also be derived from previously not annotated intergenic transcription units. We found 126 genes in which stress treatment affected alternative poly(A) sites in intergenic regions and thus regulated the expression of previously unknown poly(A)^+^ mRNA isoforms with extended 3′UTRs. Stress also influenced poly(A) site usage in the 5′UTR of the *EGLN1* (egl-9 family hypoxia-inducible factor 1) mRNA (Fig. 1B). These data show that stress treatment induces both 3′UTR- as well as CR-APA, thus regulating the length of the 3′UTRs and the open reading frames of the affected genes.

We next tested for stress-dependent differences in the regulation of promoter-proximal and -distal poly(A) sites. For each gene undergoing stress-induced APA, we separated the gene region between the most proximal and the most distal poly(A) site into five equal sections I–V and assigned all poly(A) sites that lay between the most proximal and the most distal poly(A) to the section they occurred in. In this way, we could categorize each poly(A) site dependent on its relative distance to the most proximal poly(A) site and classify it as proximal when lying closer to the most proximal poly(A) site or distal when lying closer to the most distal poly(A) site (Fig. 1C). When investigating the stress-induced regulation of the different poly(A) sites, we found that the stress-APA candidates clustered into those showing a decreased utilization of their most proximal poly(A) sites (cluster 1) and an increased utilization of their most distal poly(A) sites (cluster 2) (Fig. 1C). We grouped all genes showing the opposite trend in stress-dependent poly(A) site use into cluster 3. While 64% of the repressed proximal poly(A) sites of the cluster 1 and 2 genes occurred in ORFs or introns, we found the more frequently used distal poly(A) sites mainly in intergenic regions (36%) and 3′UTRs (49%) (Fig. 1D; Supplemental Table S2). We did not observe such a pattern in the genomic distribution of the poly(A) sites of cluster 3 genes. These data reveal that stress tends to stimulate the expression of longer and represses the expression of shorter mRNA isoforms. These results are in line with earlier studies that reported elongated 3′UTRs under cold shock in murine cells (Liu et al. 2013) and a loss of short poly(A) transcripts in yeast upon DNA damage (Graber et al. 2013).

### Stress regulates the abundance of specific alternatively polyadenylated mRNA isoforms

We next analyzed how the observed stress-induced changes in poly(A) site usage correlated with quantitative changes in total gene expression. We thus first explored transcriptome-wide changes in gene expression upon anisomycin treatment in an RNA-seq-based approach. We performed mRNA-seq on cytoplasmic RNA and pre-mRNA-seq on nuclear RNA (Supplemental Fig. S2A), which allowed us to distinguish between transcriptional and post-transcriptional effects on gene expression levels. We chose to use pre-mRNA-seq over other methods currently used to globally assess RNA transcription because it does not require any pretreatment of cells, for example with nucleotide analogs, and thus provides an unbiased in vivo snapshot of cellular pre-mRNA levels.

**FIGURE 2.**
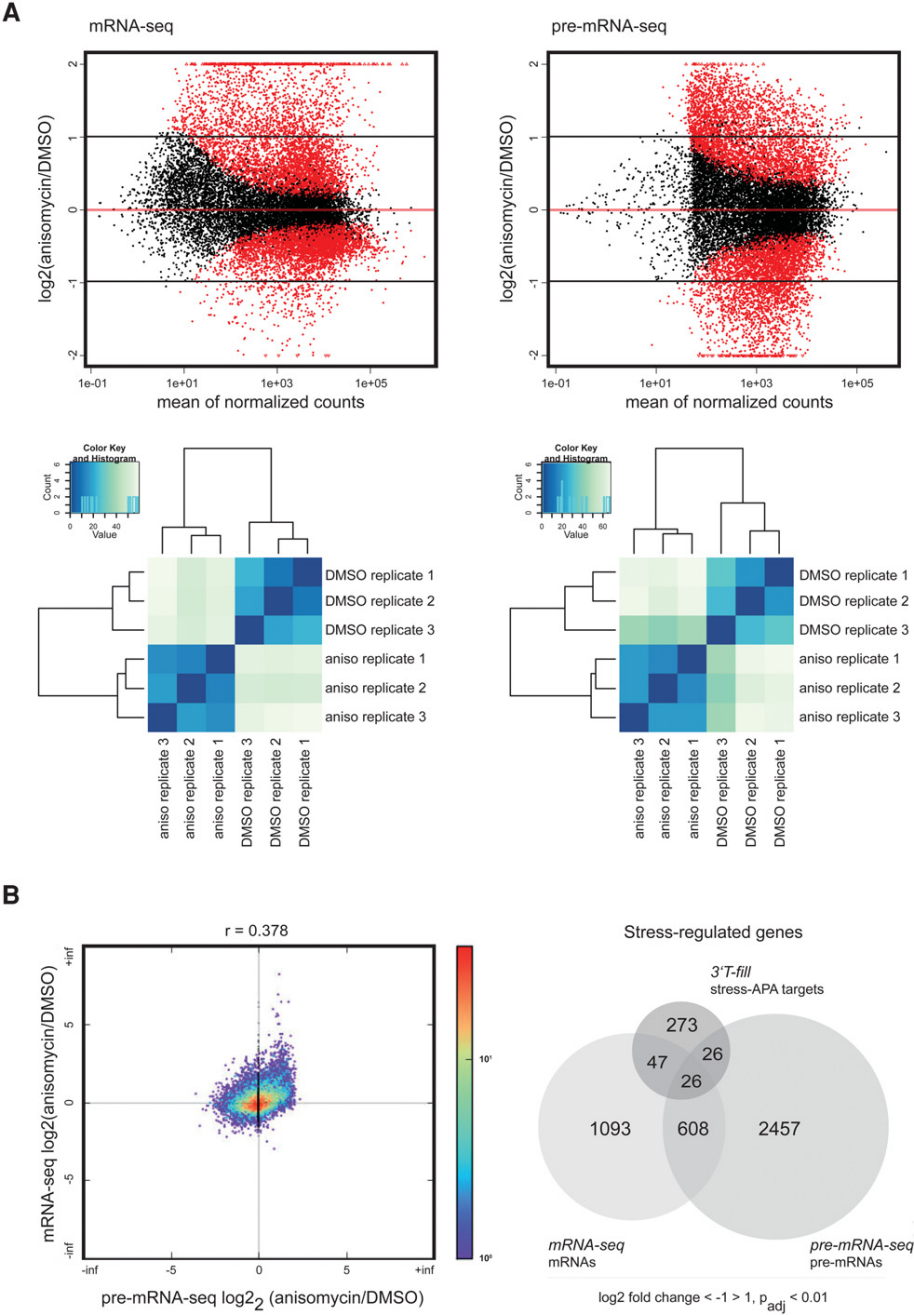
Post-transcriptional mechanisms extensively influence gene expression under stress. (*A*) Scatterplots displaying RNA-seq data show expression changes of mRNA and pre-mRNA following stress (*upper* panels). Significantly stress-regulated genes are displayed as red dots (*P*_adj_ ≤ 0.01). Genes with fold changes off the scale are shown as triangles. Only at least twofold up- or down-regulated genes (log_2_ fold-change ≥ 1 or ≤ −1) were considered for further analyses. Fold-change cutoffs are displayed as black lines. Hierarchical clustering illustrates that triplicates of mRNA-seq and pre-mRNA-seq data group according to treatment (anisomycin [“aniso”] versus control DMSO treatment) (*lower* panels). The histograms and the accompanying dendrograms display the sample-to-sample distances (Euclidean distances) between the samples which were calculated from the read counts after log transformation. (*B*) Scatterplot correlating the stress response on the pre-mRNA and the mRNA levels of coding and noncoding genes (*left* panel). Colors indicate the number of overlapping data points. Pie chart comparing absolute numbers of stress-APA targets and stress-regulated mRNAs and pre-mRNAs (*right* panel).

We controlled for the quality of the nuclear and the cytoplasmic RNA fractions by measuring *GAPDH* and *preGAPDH* levels in both nuclear and cytoplasmic RNA samples and by calculating the relative distribution of *GAPDH* and *preGAPDH* between both fractions. While we found *preGAPDH* almost exclusively in nuclear fractions, *GAPDH* mRNA was enriched in cytoplasmic RNA fractions (Supplemental Fig. S2B), which confirmed the integrity and the separation of the nuclear and the cytoplasmic RNA samples. We mapped the reads obtained in the mRNA- and the pre-mRNA-seq analyses to the human reference genome GRCh37 (Genome Reference Consortium Human Reference 37, as provided by Ensembl 73). For pre-mRNA-seq we only considered reads that included at least 7 consecutive nucleotides (nt) of intronic sequence to exclude reads that potentially derived from mature mRNA (see statistics of mRNA- and pre-mRNA-seq analyses in Supplemental Table S3). The results show that the mRNA and pre-mRNA sequencing data were highly reproducible between biological replicates (Fig. 2A).

mRNA- and pre-mRNA-seq analyses revealed that anisomycin-induced stress influenced the expression of hundreds of mRNAs and pre-mRNAs (log_2_fc ≥ 1 or ≤ −1, *P*_adj_ ≤ 0.01) (Fig. 2A; Supplemental Table S4). We compared the stress-regulated mRNA with the stress-regulated pre-mRNA levels of genes detected in both data sets to distinguish between transcriptional and post-transcriptional stress effects. Interestingly, these analyses show that 1140 (64%) of all 1774 stress-controlled RNAs that were detected both in pre- and mRNA-seq remained unchanged at the pre-RNA level, indicating that these were regulated via post-transcriptional mechanisms (Fig. 2B).

We examined the overlap between the genes that displayed stress-induced alternative polyadenylation and those that were up- or down-regulated upon stress to investigate the influence of stress-induced APA on mRNA abundance. Out of the 401 genes that underwent stress-dependent alternative polyadenylation, we detected 372 genes in both the mRNA- and pre-mRNA-seq data set. Surprisingly, only a minority of the mRNAs that were subjected to stress-induced APA showed significant alterations in overall abundance (*n* = 99) (Fig. 2B).

**FIGURE 3.**
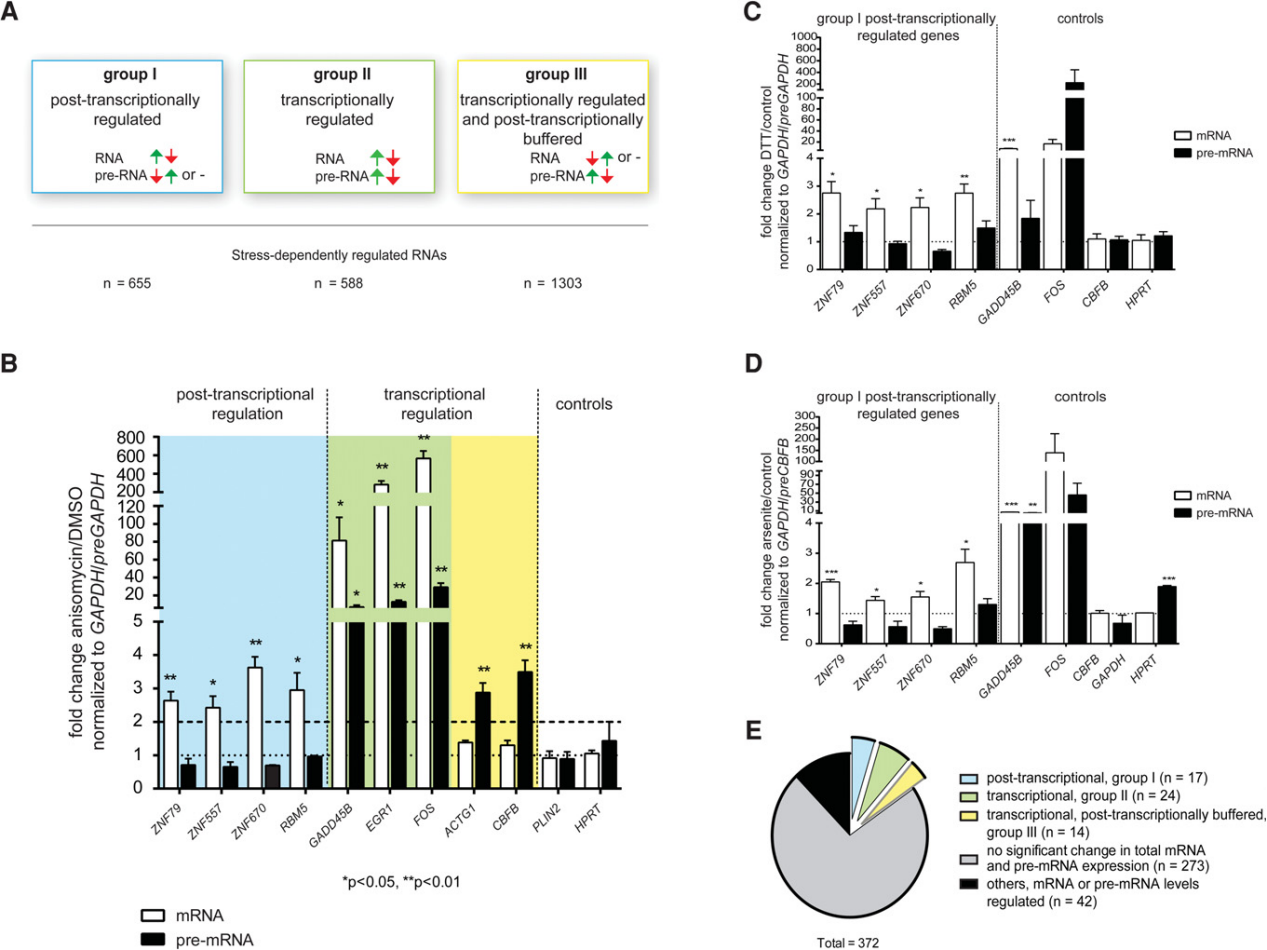
Transcriptional and post-transcriptional mechanisms target different groups of genes. (*A*) Genes were grouped according to the pattern of stress responses on the pre-mRNA and mRNA levels. Group I (blue) comprises post-transcriptionally regulated genes with regulated mRNA but unchanged or reversely regulated pre-mRNA levels. Group II (green) contains transcriptionally regulated genes with stress-induced mRNA and pre-mRNA levels. Group III (yellow) comprises genes with regulated pre-mRNA that are buffered at a post-transcriptional level and show unchanged or reversely regulated mRNA levels. (*B*) qRT-PCR validation of mRNA- and pre-mRNA-seq data of a subset of transcriptionally and post-transcriptionally up-regulated protein-coding genes. mRNA and pre-mRNA levels of genes randomly selected from group I, II, and III were compared to control genes (*CBFB*, *HPRT*). Data points represent mean + SEM of ≥ 3 independent qRT-PCR experiments normalized to *GAPDH* or *preGAPDH*, respectively. Two-sided Student's *t*-test was used to calculate *P*-values. (*C*,*D*) qRT-PCRs measuring mRNA and pre-mRNA levels of candidate genes (*ZNF79*, *ZNF557*, *ZNF670*, and *RBM5*) and not post-transcriptionally regulated control mRNAs (*GADD45B*, *FOS*, *CBFB*, and *HPRT*) after DTT (*C*) and arsenite treatment (*D*). Data were normalized to unstressed cells and *GAPDH* or *preGAPDH* in DTT-treated cells. Because arsenite treatment influenced *GAPDH* pre-mRNA levels, pre-mRNA data were normalized to *preCBFB* in arsenite-treated cells. Bars represent mean + SEM, *n* ≥ 3. Two-sided Student's *t*-test was applied to calculate *P*-values. (*E*) Genes undergoing stress-induced APA were analyzed for total gene expression changes in stress and categorized into the groups I (blue), II (green), or III (yellow). The remaining genes either did not show a significant change in mRNA and pre-mRNA expression in stress (gray) or did not belong to any of the mentioned categories (black). These included genes in which stress treatment affected both mRNA and pre-mRNA expression but significantly regulated (*P*_adj_ < 0.01) only mRNA levels or vice versa.

In order to accurately distinguish at which level stress-induced APA influenced mRNA abundance, we classified stress-regulated genes according to their stress response patterns into three groups (Fig. 3A). Group I includes post-transcriptionally regulated genes with significantly (*P*_adj_ ≤ 0.01) down- or up-regulated mRNA levels and unchanged or reversely regulated pre-mRNA levels (log_2_-fold change _pre-mRNA_ ≥ −0.38 or ≤ 0.38). In this way, we excluded all genes that were significantly regulated on the mRNA level and showed a strong trend toward concordant changes in pre-mRNA levels from our downstream analyses ensuring that group I only contains high confident post-transcriptionally regulated mRNAs. Group II contains transcriptionally regulated genes that were significantly (*P*_adj_ ≤ 0.01) and at least twofold down- or up-regulated at the pre-mRNA level with correspondingly regulated mRNA levels. Group III genes show significantly (*P*_adj_ ≤ 0.01) down- or up-regulated pre-mRNA but unchanged or reversely regulated mRNA levels (log_2_-fold change _mRNA_ ≥ −0.38 or ≤ 0.38), thus indicating that changes in pre-mRNA levels were not mirrored at the mRNA level in these cases. Again, all genes showing a significant change in their pre-mRNA and a detectable but not significant concordant regulation of their mRNA levels were excluded from group III and thus downstream analyses. Control genes displayed no changes to stress at the mRNA or pre-mRNA level. We validated a selection of group I, II, and III genes that were up-regulated in response to anisomycin treatment by qRT-PCR using primers specific for mRNA and pre-mRNA (Fig. 3B).

We detected a post-transcriptional stimulation of selected group I genes also in DTT-induced ER-stress and arsenite-induced oxidative stress (Fig. 3C,D), which indicated that different forms of cellular stress influence mRNA expression by post-transcriptional mechanisms.

We next categorized the genes undergoing stress-induced APA into the groups I, II, and III. The APA targets whose abundance changed in a stress-dependent fashion either belonged to group I (*n* = 17) with their total expression levels being post-transcriptionally regulated, group II (*n* = 24) or group III (*n* = 14) with their total expression levels being transcriptionally regulated in stress (Fig. 3E; Supplemental Table S5). In those, stress-induced APA might thus have an impact on overall mRNA abundance.

### Stress-induced differential expression of polyadenylated isoforms influences the abundance of specific mRNAs

Since stress-dependent APA should act post-transcriptionally, we focused on the group I APA targets, which included 15 genes with up- and two with down-regulated total mRNA levels. We investigated a subset of five group I genes and found that the observed changes in mRNA abundance upon stress treatment were not caused by stress-induced mRNA stabilization (Supplemental Fig. S3), indicating that these rather resulted from the differential usage of poly(A) sites.

**FIGURE 4.**
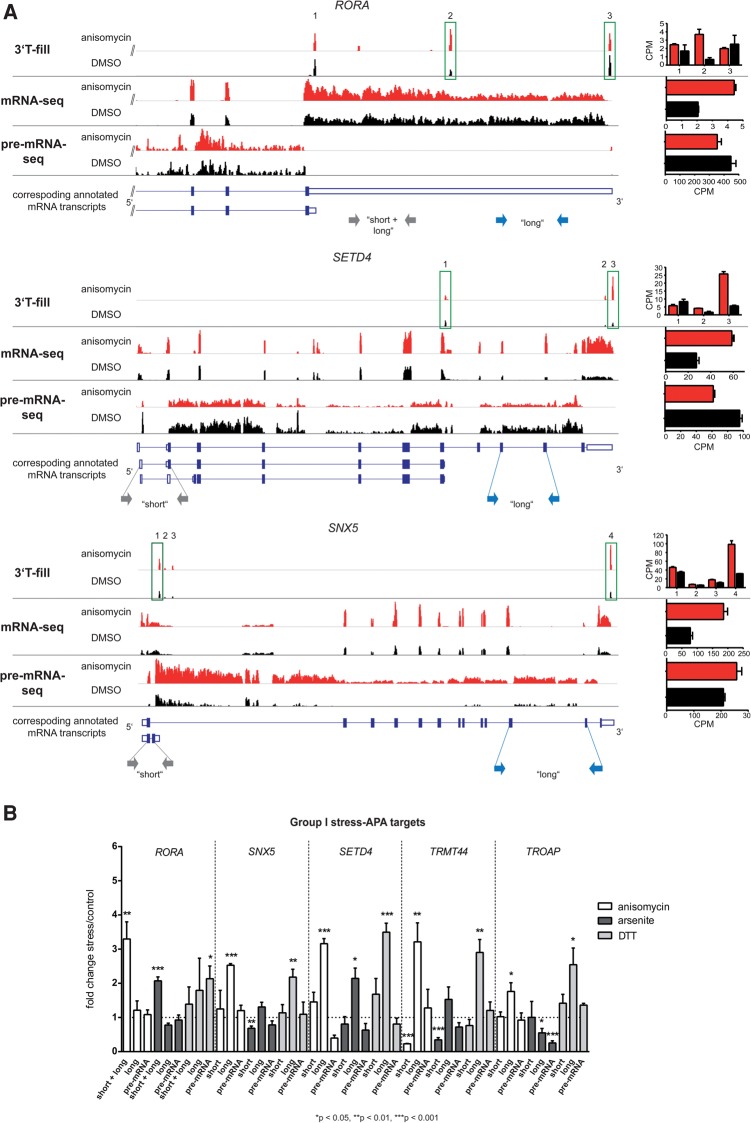
Stress-induced APA can quantitatively modulate the expression of distinct mRNA isoforms. (*A*) IGV browser data displaying the 3′T-fill, mRNA, and pre-mRNA-seq data for the genes *RORA* (*upper* panel), *SETD4* (*middle* panel), and *SNX5* (*lower* panel) in stress (red) and control conditions (black) as examples for group I APA targets. The 3′T-fill lanes show poly(A) sites used under stressed (anisomycin) and unstressed (DMSO) conditions. Detected poly(A) sites are numbered and poly(A) sites that are significantly regulated relative to other poly(A) sites within the same gene are highlighted with green boxes. The mRNA-seq lanes display the expression of poly(A)^+^ mRNA and the pre-mRNA-seq lanes the expression of intronic regions. The *bottom* lanes schematically represent the mRNA transcripts of the respective genes as provided by Ensembl 73 with coding exons as filled boxes, 3′UTRs as open boxes, and introns as lines. The bar diagrams on the *right* display the mean 3′T-fill expression data for individual poly(A) sites (*upper* panel) and the quantification of total mRNA (*middle* panel) or pre-mRNA levels (*lower* panel) as obtained in mRNA- and pre-mRNA-seq sequencing analyses of three independent biological replicates. The gray and blue arrows indicate the primers used in the qRT-PCR displayed in panel C to specifically amplify the “short,” the “long,” or both (“short + long”) mRNA isoforms of the respective genes*.* (*B*) qRT-PCRs on cells treated with anisomycin, arsenite, or DTT using primers specific for the “short,” the “long,” or both (“short + long”) isoforms of the genes *RORA*, *SETD4*, *SNX5, TRMT44*, and *TROAP.* Pre-mRNA levels were assessed to confirm that changes in gene expression happened post-transcriptionally. Data were normalized to unstressed cells and *GAPDH* or *preGAPDH* in DTT- and anisomycin-treated cells. Because arsenite treatment influenced *GAPDH* pre-mRNA levels, pre-mRNA data were normalized to *preCBFB* in arsenite-treated cells. Bars represent mean + SEM, *n* ≥ 3. Two-sided Student's *t*-test was applied to calculate *P*-values.

In five group I APA targets, including *RORA* (RAR-Related Orphan Receptor A), *SETD4* (SET Domain Containing 4) and *SNX5* (Sorting Nexin 5), stress treatment induced a switch in main poly(A)^+^ isoform expression. Specifically, mapping of the 3′ ends of the *RORA* mRNA by 3′T-fill revealed that anisomycin up-regulated the expression of an mRNA with a not annotated poly(A) site occurring in the 3′UTR between two known poly(A) sites ∼4 kb downstream and ∼5 kb upstream, respectively. This up-regulation is also reflected by the stress-induced difference in read numbers in the mRNA-seq data upstream and downstream of the previously unannotated poly(A) site (Fig. 4A). In the genes *SETD4* and *SNX5*, stress specifically stimulated the use of poly(A) sites corresponding to the long annotated mRNA isoforms with extended open reading frames (ORFs). As for *RORA*, this is also reflected by the difference in read numbers of the short and the long mRNA isoforms of the genes in the mRNA-seq (Fig. 4A).

We could validate the 3′T-fill results of *RORA*, *SETD4*, and *SNX5* as well as of the other group I genes *TRMT44* (TRNA methyltransferase 44 homolog [*Saccharomyces cerevisiae*]) and *TROAP* (Trophinin-associated protein) in qRT-PCRs with isoform-specific primers and found that these genes undergo APA under various stress conditions (Fig. 4B). Further to anisomycin-induced ribostress, *SNX5*, *SETD4, RORA, TRMT44,* and *TROAP* underwent APA in arsenite-induced oxidative stress and all genes except *RORA* were also subjected to APA in DTT-induced ER stress.

As we have also observed on a global scale (see above), stress led to a decreased utilization of proximal poly(A) sites and/or an increased utilization of distal poly(A) sites in 10 out of 15 up-regulated group I APA genes indicating that, in contrast to other studies performed on proliferating and cancer cells (Sandberg et al. 2008; Mayr and Bartel 2009), mRNA shortening does not correlate with up-regulated expression levels of these genes. Likewise, the two down-regulated genes *CLN8* and *WDR6* showed down-regulated elongated mRNA isoforms, suggesting that stress-induced modifications in mRNA length do not inversely correlate with stress-dependent changes in mRNA abundance.

Our results show that various forms of stress can influence the utilization of poly(A) sites and the overall mRNA abundance of a small group of genes, including *RORA*, *SETD4*, *SNX5*, *TRMT44,* and *TROAP*.

### Stress-induced APA extensively affects mRNA configuration

**FIGURE 5.**
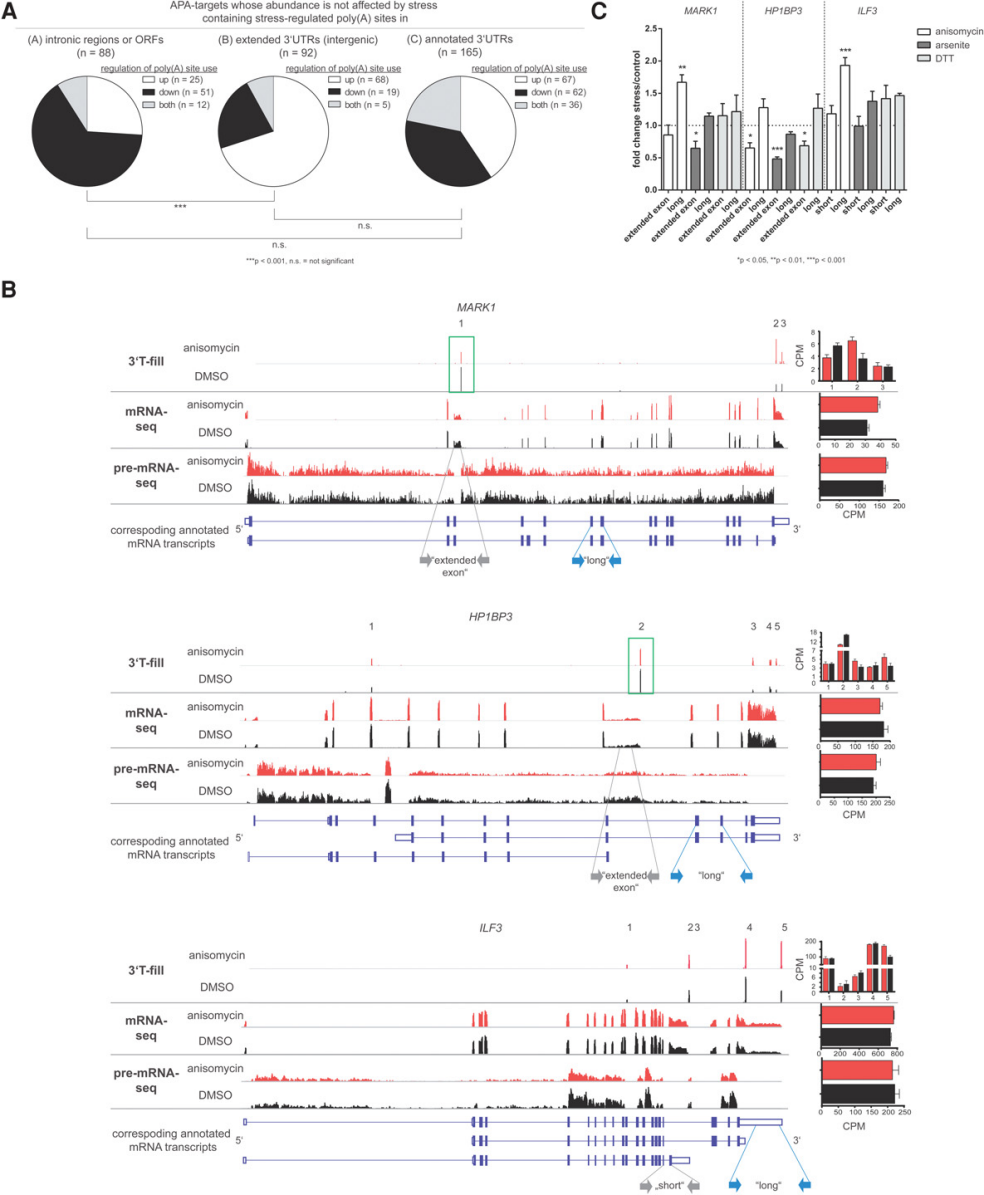
Stress-induced APA regulates the length of the ORF of genes whose mRNA abundance is not affected by stress treatment. (*A*) Pie charts displaying the proportion of genes with stress-dependently regulated poly(A) sites in (*A*) intronic regions or ORFs, (*B*) intergenic regions (extended 3′UTRs), or (*C*) annotated 3′UTRs among the APA targets that do not change in overall gene expression in stress. Genes may be included in more than one category if possessing multiple stress-regulated poly(A) sites in different genomic regions. Genes containing poly(A) sites which showed an increased usage are depicted in white, those containing poly(A) sites which showed a decreased usage are shown in black, and those containing both are shown in gray. χ^2^ test was applied to calculate *P*-values. (*B*) IGV browser data displaying the 3′T-fill, mRNA- and pre-mRNA-seq data for the genes *MARK1* (*upper* panel), *HP1BP3* (*middle* panel), and *ILF3* (*lower* panel) in stress (red) and control conditions (black). The 3′T-fill lanes show poly(A) sites used under stressed (anisomycin) and unstressed (DMSO) conditions. Detected poly(A) sites are numbered. Poly(A) sites corresponding to previously not annotated isoforms are highlighted with green boxes. The mRNA-seq lanes display the expression of poly(A)^+^ mRNA and the pre-mRNA-seq lanes the expression of intronic regions. The bar diagrams on the *right* display the 3′T-fill expression data for individual poly(A) sites (*upper* panel) and the quantification of total mRNA (*middle* panel) or pre-mRNA levels (*lower* panel) as obtained in mRNA- and pre-mRNA-seq sequencing analyses. The *bottom* lanes schematically represent the mRNA transcripts of the respective genes as provided by Ensembl 73 with coding exons as filled boxes, 3′UTRs as open boxes, and introns as lines. The gray and blue arrows indicate the primers for the short isoform of *ILF3*, the isoforms with an “extended exon” of *MARK1* and *HP1BP3* or the annotated “long” isoforms that were used in the qRT-PCR displayed in panel *C*. (*C*) qRT-PCRs on cells treated with anisomycin, arsenite, or DTT using primers specific for the annotated “long” or “short” isoforms or the isoforms with an “extended exon” (see panel *B*) of the genes *MARK1, HP1BP3,* and *ILF3*. Data were normalized to unstressed cells and *GAPDH*. Bars represent mean + SEM, *n* = 3. Two-sided Student's *t*-test was applied to calculate *P*-values.

Interestingly, 273 genes undergoing stress-induced APA did not significantly change in total mRNA or pre-mRNA abundance (Fig. 3E). APA might thus influence these genes beyond regulating their overall abundance as has been also reported in earlier studies on individual genes that showed APA-driven modifications to modulate cellular mRNA function without affecting total expression levels (Berkovits and Mayr 2015; Vilborg et al. 2015). To unravel the biological significance of APA in these 273 genes on a global scale we asked how poly(A) site usage in these RNAs is regulated depending on the genomic localization of the stress-regulated poly(A) sites. While the utilization of stress-regulated poly(A) sites that we identified in annotated 3′UTRs was increased in ∼41% and decreased in ∼38% of the genes thus not showing a clear preference into either direction, we observed a significant difference in stress-induced regulation between poly(A) sites in intergenic regions and poly(A) sites in introns/ORFs (Fig. 5A). We observed that stress treatment strongly stimulated the usage of intergenic polyadenylation sites and found the utilization of stress-regulated intergenic poly(A) sites to be increased in ∼74% of the genes that contained such poly(A) sites, which stimulated the expression of 3′ extended mRNA isoforms. In contrast, stress repressed the utilization of poly(A) sites in intronic regions or ORFs. In ∼57% of the genes that possessed stress-regulated poly(A) sites in introns or ORFs, these sites were up-regulated upon stress treatment (Fig. 5A). These results are in line with the trend in poly(A) site usage we have detected earlier when investigating a subset of APA targets with two PAS (Fig. 1C). We observed that the repression of alternative poly(A) site usage in intronic regions or ORFs often led to the preferred expression of longer mRNA isoforms with different coding potential. This is exemplified by the genes *MARK1* (MAP/microtubule affinity-regulating kinase 1)*, HP1BP3* (Heterochromatin Protein 1), and *ILF3* (Interleukin enhancer binding factor 3, 90 kDa), where the 3′T-fill analysis revealed that stress differentially regulated *MARK1, HP1BP3,* and *ILF3* transcripts with alternative ORFs (Fig. 5B). In all three cases, stress-induced APA stimulated the expression of the full-length mRNA isoforms of these genes. We confirmed the stress-regulated change in poly(A) site usage for the *MARK1, HP1BP3,* and *ILF3* mRNA isoforms by qRT-PCRs (Fig. 5C). In addition, we found that the *MARK1* and *HP1BP3* genes undergo CR-APA in response to different cellular stresses (Fig. 5C). While *MARK1* showed a switch in poly(A) site usage upon treatment with anisomycin and arsenite, *HP1BP3* shows a differential expression of its long and its short mRNA isoforms upon exposure to anisomycin, arsenite, and DTT.

These data reveal that stress-induced APA extensively influences mRNA configuration and thus regulates gene expression beyond affecting mRNA abundance. We find that stress-dependent APA decreases the usage of poly(A) sites within ORFs and introns and increases the utilization of intergenic poly(A) sites, which stimulates the expression of full-length, and in many cases 3′ extended, mRNA isoforms.

## DISCUSSION

We demonstrate that alternative polyadenylation contributes to the post-transcriptional stress response in a previously unrecognized fashion and affects a large number of mRNAs and ncRNAs. Applying stringent filtering criteria, we identify 401 genes that undergo stress-induced APA and find that stress treatment induces 3′UTR- or CR-APA in these genes. We detect stress to regulate the usage of poly(A) sites within annotated 3′UTRs (3′UTR-APA) in 248 genes, which modifies the 3′ ends of these transcripts. This may either affect 3′UTR length (potentially determining the inclusion or exclusion of *cis*-acting RNA sequence elements such as miRNA binding sites or binding sites for regulatory RNA-binding proteins) (Hollerer et al. 2014) or influence the expression of annotated isoforms with different ORFs. In addition, we identified 138 genes with stress-regulated poly(A) sites in intronic and coding regions thus undergoing CR-APA. We further detected 126 genes in which stress-regulated poly(A) sites occurred in intergenic regions downstream from annotated gene regions thus likely corresponding to 3′ elongated mRNA isoforms of these genes.

We find a remarkable difference in regulation when comparing proximal and distal poly(A) sites and observe a global trend toward the stimulated utilization of distal and the repressed utilization of proximal poly(A) sites. A similar trend has been reported in mice upon cold shock (Liu et al. 2013) and yeast upon DNA damage (Graber et al. 2013). Our data thus suggest the stress-dependent inhibited usage of proximal and the increased usage of distal poly(A) sites as a general stress response mechanism.

When analyzing the alternatively polyadenylated genes by mRNA- and pre-mRNA-seq, we found that the abundance of most APA targets remained unchanged in stress. These data show that stress-induced APA mainly affects gene expression in a qualitative manner, which contrasts to proliferating and cancer cells in whom APA was reported to have a major effect on mRNA and protein abundance (Sandberg et al. 2008; Mayr and Bartel 2009). Our small set of stress-APA targets with altered total RNA and/or pre-RNA abundance included protein-coding and noncoding genes whose expression was post-transcriptionally (group I) or transcriptionally regulated (group II and III) upon stress treatment, including *SETD4* and *SNX5* for which the differential expression of mRNA isoforms changes the ORF, as well as *RORA* where stress triggers the expression of an mRNA isoform with a shortened 3′UTR. The group of APA targets whose overall abundance changes in stress comprises genes exerting numerous cellular functions, ranging from intracellular trafficking (*SNX5*), signaling (*IQGAP1*) to transcriptional regulation (*RORA, TAF1D*). Some of these genes are known to play a role in stress, such as *RORA* and *AP5Z1,* which have been reported to be involved in the DNA damage response (Slabicki et al. 2010; Kim et al. 2011), or *SETD4*, whose expression is induced upon exposure to cigarette smoke (Sundar et al. 2014). Future studies will focus on exploring how APA affects the stress-dependent function of these genes.

Interestingly, ∼73% of the genes undergoing stress-induced APA stay unchanged in total mRNA and pre-mRNA expression levels showing that stress-induced APA mainly influences the gene expression pathway without affecting mRNA abundance. This is in line with other studies that reported APA-dependent modifications in 3′UTR length to influence expression to a minor extent (Spies et al. 2013; Gruber et al. 2014; Gupta et al. 2014). Differences in 3′UTR length provoked by 3′UTR-APA can, however, influence important functions of the mRNA without necessarily changing mRNA abundance, as has recently been highlighted by the finding that cellular proteins can bind to the 3′UTR and thereby determine protein localization (Berkovits and Mayr 2015). Similarly, regulating CR-APA events potentially leads to the expression of noncoding RNAs with stress-specific functions or can change the coding potential of these genes, as is the case for immunoglobulins during the heavy chain (IgH) class shift (Brown and Morrison 1989). We find that the stress-regulated poly(A) sites of the mRNAs whose abundance remains unchanged are differentially regulated depending on their genomic localization. While poly(A) sites occurring in annotated 3′UTRs are equally up- and down-regulated, intergenic poly(A) sites show an enhanced utilization under stress conditions in 74% of all cases. A recently published study found the expression of such 3′ elongated transcripts to be also induced by osmotic stress (Vilborg et al. 2015), which indicates that the observed stress-dependent up-regulated utilization of intergenic polyadenylation may constitute a response that is common to different forms of stress. In contrast, stress conditions repressed the use of most poly(A) sites localized in coding regions, which might also be a result of alternative splicing as has been reported in recent studies (Almada et al. 2013; Li et al. 2015). We observe that CR-APA inhibits the expression of “truncated” while stimulating the expression of full-length mRNA isoforms in many cases. The functional potential of this differential expression of mRNA isoforms is exemplified by the microtubule affinity-regulated kinase 1 (*MARK1*), the heterochromatin protein 1 binding protein 3 (*HP1BP3*) and the interleukin enhancer binding factor 3, 90 kDa (*ILF3*) genes. *MARK1* encodes a kinase that is known to phosphorylate microtubule-associated proteins and triggers microtubule disruption (Drewes et al. 1997). Interestingly, only the full-length ORF that is induced by stress contains the kinase and a kinase-associated domain (KA1) as well as a ubiquitin-associated domain (UBA), suggesting that this longer isoform exerts a stress-specific function (Supplemental Fig. S4).

HP1BP3 is a protein involved in cell cycle control and mediates chromatin condensation under hypoxic conditions leading to increased tumor cell viability, radio-resistance, chemo-resistance, and self-renewal (Dutta et al. 2014a,b). Similarly to *MARK1*, only the stress-induced full-length mRNA isoform codes for a fully functional protein including three H15 domains that play an essential role in nucleosome binding (Supplemental Fig. S4).

The *ILF3* gene encodes the 90-kDa protein dsRNA-binding subunit of the transcription factor NFAT. The protein product of the stress-induced longer isoform of the subunit harbors a fully functional PRMT1 interaction domain, which is lacking in the short non-stress-regulated mRNA isoform. With this domain, ILF3 can interact and regulate the protein-arginine methyltransferase PRMT1 (Tang et al. 2000), an enzyme that is involved in several critically cellular pathways, including signal transduction, transcription, DNA repair and splicing but also carcinogenesis and metastasis (Supplemental Fig. S4; Yang and Bedford 2013).

The stress-induced differential expression of alternatively polyadenylated mRNA isoforms can thus lead to the inclusion or exclusion of functional domains. Consistent with the known inhibition of translation under conditions of cellular stress (Ron and Harding 2007), we have observed suppressed protein synthesis following anisomycin, DTT, and arsenite treatment (Supplemental Fig. S5). We thus hypothesize that the synthesis of the protein isoforms encoded by these alternatively polyadenylated transcripts may play a role in the recovery phase following stress, which will be interesting to address in model systems.

In sum, our study uncovers alternative polyadenylation as a widespread stress response mechanism that contributes to the complexity of stress-induced post-transcriptional gene regulation by extensively influencing gene expression in a qualitative manner. We find that stress-induced APA regulates gene expression mainly by influencing mRNA configuration. Our data reveal a global trend in the stress-dependent usage of poly(A) sites towards the repression of poly(A) site usage within introns and ORFs and the increased usage of distal poly(A) sites, stimulating the expression of full-length, often 3′ extended, mRNA isoforms.

## MATERIALS AND METHODS

### Cell culture

Flp-In T-REx 293 cells (#R780-07, Invitrogen) were cultivated at 37°C with 5% CO_2_ in Dulbecco's modified Eagle's medium (DMEM) with 10% fetal bovine serum (FBS) (Life Technologies) and antibiotic (100 U/mL penicillin, 100 µg/mL streptomycin, 200 µg/mL Zeocin, and 15 µg/mL blasticidin S).

### Stress experiments

Cells were seeded 48 h prior to stress treatment. Cells were treated with 5 µg/mL anisomycin (Calbiochem), 100 µM arsenite (Sigma), or 3 mM DTT (Promega) for 3 h and harvested in ice-cold PBS.

### RNA extraction

For RNA-seq and validation experiments, cells were fractionated and cytoplasmic and nuclear RNA were isolated using the PARIS Kit (Ambion) to enrich for the respective RNA species. gDNA was depleted on gDNA eliminator columns (QIAGEN) and RNA was purified with the RNeasy Mini kit (QIAGEN). For DTT and arsenite experiments, total RNA was isolated using TRIzol LS.

### qRT-PCR

Real-time PCR was used to quantify mRNA and pre-mRNA levels. For reverse transcription of mRNA and pre-mRNA, cDNA synthesis was carried out with oligo(dT) primers or random hexamers, respectively, using RevertAid H Minus Reverse Transcriptase (Thermo scientific) according to the manufacturer's instructions. mRNA and pre-mRNA levels were quantified using a StepOnePlus Real-time PCR system (Life Technologies).

### mRNA stability assays (pulse chase experiments)

Newly synthesized RNAs were labeled by treating cells with 100 µM ethylene uridine (EU, Life Technologies) for 2 h at 37°C. The EU-label was washed out and cells were subsequently treated with 5 µg/mL anisomycin or DMSO. Transcription was simultaneously blocked by adding 60 µM DRB (5,6-dichloro-1-β-D-ribofuranosylbenzimidazole, Sigma-Aldrich). Cells were harvested before (*t* = 0) and after 3 h of stress treatment. EU-labeled RNA was purified with the Click-it Nascent RNA Capture kit (Life Technologies) according to the manufacturer's instructions, and EU-RNA levels were determined on a StepOnePlus Real-time PCR system (Life Technologies).

### ^35^S-methionine assay

For capturing de novo synthesized proteins, cells were treated with 5 µg/mL anisomycin, 3 mM DTT, 100 µM arsenite, or the solvent control DMSO or were left untreated. De novo translated proteins were labeled by adding DMEM -Met, -Cys, -Glu (Gibco) that contained 10% FBS (dialyzed, Sigma-Aldrich), 1× GlutaMax (Gibco) and ∼20 µCi/mL ^35^S-Met (-Met-35S-Label, Hartmann Analytic). Cells were harvested in PBS and lysed in RIPA buffer containing protease inhibitor (cOmplete Protease Inhibitor Cocktail, Roche). Protein lysates were spotted onto glass microfiber filters (Whatman) and proteins were precipitated in 15% ice-cold trichloroacetic acid. ^35^S counts were measured in a scintillation counter.

### Pre-mRNA- and mRNA-seq library preparation

Nuclear RNA samples were depleted from ribosomal RNA using the Ribo-Zero Gold Kit (Epicenter). Pre-mRNA- (from nuclear RNA) and mRNA-seq libraries (from cytoplasmic RNA) were created according to standard Illumina TruSeq protocols excluding or including the initial poly(A)^+^ mRNA purification step.

### Sequencing

All samples were clustered and sequenced on a HiSeq2000 (Illumina). mRNA- and pre-mRNA-seq libraries were subjected to single-end sequencing. Read lengths were 50 bp.

### Read preprocessing and alignment

mRNA- and pre-mRNA-seq reads were trimmed to remove adapter sequences. Reads >20 bp were mapped to the human reference genome GRCh37 (Genome Reference Consortium Human Reference 37, as provided by Ensembl 73) with Bowtie. Reads per annotated gene were counted using HTSeq (Anders et al. 2015). DESeq2 (Love et al. 2014) was applied to investigate differences in mRNA expression in anisomycin- and DMSO-treated control T-REx 293 cells. In pre-mRNA-seq, only reads overlapping an intron by at least 7 nt were kept to exclusively capture pre-mRNAs. For downstream analysis, low abundant targets with less than 1 count per million sequenced reads (CPM) were filtered out in both mRNA- and pre-mRNA-seq data sets.

### Differential expression analysis

Differentially expressed genes between stress and control conditions were determined with DESeq2 version 1.2.8 following the protocol published by Anders et al. (2013).

### 3′T-fill library preparation and sequencing

3′T-fill was performed on three independent biological replicates as described by Wilkening et al. (2013). In brief, cytoplasmic RNA was fragmented and reverse transcribed with an oligo(dT) primer ligated to a biotin-coupled adapter. After second strand cDNA-synthesis, fragments were bound to beads and a barcoded adapter was added to the captured fragment. Poly(A) tails were saturated with unlabeled dTTPs on the cluster station before sequencing started directly at the end of the 3′UTR, with the first base sequenced representing the poly(A) site. 3′T-fill libraries were subjected to paired-end sequencing with a read length of 50 bp.

### Definition of high-confidence poly(A) sites

3′T-fill libraries were demultiplexed according to their barcodes (see Supplemental Table S6). Sequencing adapters and poly(A) tails were removed by trimming. Reads containing more than 80% A- and T-residues were removed. Sequencing reads >20 bp were mapped to the human reference genome GRCh37 (Genome Reference Consortium Human Reference 37, as provided by Ensembl 73) with Bowtie. Internal priming events were defined as described in previous studies: Reads with six continuous A's or more than seven A's within the first 10 sequenced nucleotides were removed (Tian et al. 2005). However, internal priming candidates were considered for further analysis when containing the polyadenylation hexamer AAUAAA or one of its 11 variants −40 to −1 nt upstream of the detected poly(A) site (Beaudoing et al. 2000). Reads were mapped to annotated transcripts. Those occurring in intergenic regions were thereby aligned to the closest upstream transcript. HTSeq (Anders et al. 2015) was used to count reads per transcript. Poly(A) sites were defined as sites supported by a minimum of 10 reads in at least one of the conditions (stress or control) and had to appear in all three biological replicates of a given condition. Counts were summed in a 100-bp window and poly(A) sites were assigned to the center of the windows. Differential poly(A) site usage within a transcript was analyzed with DEXSeq (Anders et al. 2012) comparing anisomycin- and DMSO-treated control cells.

## DATA DEPOSITION

The 3′T-fill, pre-mRNA-, and mRNA-seq data from this publication have been submitted to the ArrayExpress database (http://www.ebi.ac.uk/arrayexpress) and assigned the identifier E-MTAB-3585.

## SUPPLEMENTAL MATERIAL

Supplemental material is available for this article.

## Supplementary Material

Supplemental Material
