# Supplementary material for: The differential expression of alternatively polyadenylated transcripts is a common stress-induced response mechanism that modulates mammalian mRNA expression in a quantitative and qualitative fashion
Source: RNA. 2016 Sep;22(9):1441–53. doi: 10.1261/rna.055657.115 (PMC4986898; doi:10.1261/rna.055657.115)
Supplement: Supplemental Material [file supp_22_9_1441__index.html]

The differential expression of alternatively polyadenylated transcripts is a common stress-induced response mechanism that modulates mammalian mRNA expression in a quantitative and qualitative fashion — Supplemental Material 

# The differential expression of alternatively polyadenylated transcripts is a common stress-induced response mechanism that modulates mammalian mRNA expression in a quantitative and qualitative fashion

## Supplemental Material

**Files in this Data Supplement:**

- Supplemental\_Tables\_S2-S7.pdf
- Supp Figures.pdf
- Supplemental\_Table\_S1.xlsx
