# Supplementary material for: The differential expression of alternatively polyadenylated transcripts is a common stress-induced response mechanism that modulates mammalian mRNA expression in a quantitative and qualitative fashion
Source: RNA. 2016 Sep;22(9):1441–53. doi: 10.1261/rna.055657.115 (PMC4986898; doi:10.1261/rna.055657.115)
Supplement: Supplemental Material [file supp_055657.115_Supplemental_Tables_S2-S7.pdf]

Supplemental Table S2. Stress-dependent regulation of poly(A) site usage in cluster 1 + 2 genes depending on genomic position of poly(A) sites

	**cluster 1 + 2 genes with two poly(A) sites** showing	
	decreased proximal poly(A) site usage (N = 84)	increased distal poly(A) site usage (N = 85)
in introns/ORFs:	54 (64%)	12 (14%)
in 3'UTRs:	28 (33%)	42 (49%)
in intergenic regions:	2 (2%)	31 (36%)

Supplemental Table S3. pre-mRNA- and mRNA-Seq statistics

**sample**	**total reads**	**kept after adapter trimming**	**mapped as single hits**	**filtered for intronic reads**
**mRNA-Seq**				
DMSO R1	198,919,396	195,581,294	159,763,063	-
DMSO R2	182,102,225	178,930,835	146,293,199	-
DMSO R3	199,638,733	196,421,025	160,751,565	-
anisomycin R1	183,546,512	180,406,011	150,488,532	-
anisomycin R2	132,394,033	130,094,132	106,578,196	-
anisomycin R3	176,906,480	173,861,056	144,849,784	-
**pre-mRNA-Seq**				
DMSO R1	148,911,912	135,583,113	116,065,841	53,990,812
DMSO R2	205,628,831	202,625,639	157,191,806	65,154,190
DMSO R3	204,217,223	201,447,477	160,340,013	72,625,086
anisomycin R1	138,278,343	128,335,038	104,651,019	43,147,560
anisomycin R2	209,695,388	206,490,248	150,091,187	49,247,980
anisomycin R3	200,588,152	197,720,632	155,464,010	57,119,032

Supplemental Table S4. RNAs regulated upon anisomycin treatment
log2 fold change $\leq$ -1/ $\geq$ 1; $p_{adj} \leq 0.01$

	upregulated	downregulated
**mRNA**	1,631	278
**pre-mRNA**	1,718	1,401

Supplemental Table S5. Group I, II and III genes undergoing stress-induced APA

gene name	detected poly(A) sites in 3'T-fill			
	chromosome	chromosomal position	genomic region	DEXSeq $\log_2$ fold change anisomycin/DMSO
**Group I posttranscriptionally regulated genes**				
AFG3L1P	chr16	90051109	intron/ORF	-0.41*
	chr16	90062968	intron/ORF	0.14
	chr16	90063028	intron/ORF	0.20
AP5Z1	chr7	4831381	3‘UTR	-0.22*
	chr7	4834026	intergenic	0.86*
CLN8	chr8	1728933	3‘UTR	0.27
	chr8	1730864	3‘UTR	0.13
	chr8	1730956	3‘UTR	-0.07
	chr8	1734735	3‘UTR	-0.78*
FNBP4	chr11	47738068	intergenic	0.41*
	chr11	47765464	intron/ORF	-0.84*
IQGAP1	chr15	90934188	intron/ORF	-0.57*
	chr15	91044784	3‘UTR	0.37
	chr15	91045470	3‘UTR	0.19*
PLEKHA7	chr11	16798843	intergenic	0.58
	chr11	16996186	intron/ORF	-0.23*
PPP4R1L	chr20	56803997	intergenic	0.57*
	chr20	56820801	3‘UTR	-0.45*
RORA	chr15	60780483	3‘UTR	-0.54*
	chr15	60785330	3‘UTR	0.79*
	chr15	60789458	3‘UTR	-0.11
RP11-37B2.1	chr8	90733133	intron/ORF	0.97*
	chr8	90736717	intron/ORF	-0.37*
SETD4	chr21	37406838	3‘UTR	0.42*
	chr21	37407241	3‘UTR	0.14
	chr21	37415982	intron/ORF	-0.68*
SNX5	chr20	17922244	3‘UTR	0.23*
	chr20	17947568	intron/ORF	-0.07
	chr20	17948014	intron/ORF	-0.22
	chr20	17948344	intron/ORF	-0.14*
TAF1D	chr11	93459823	intergenic	-0.05
	chr11	93463367	3‘UTR	0.12
	chr11	93463446	3‘UTR	0.04
	chr11	93469098	3‘UTR	-1.05*
	chr11	93471471	3‘UTR	0.08
TRMT44	chr4	8458268	intron/ORF	-0.70*
	chr4	8470041	intron/ORF	0.52
	chr4	8478281	intron/ORF	0.27*
TROAP	chr12	49718152	3‘UTR	-0.52*
	chr12	49725513	3‘UTR	0.16*
WDR6	chr3	49044547	3‘UTR	0.20*
	chr3	49053384	3‘UTR	-0.12*
ZNF37BP	chr10	43009055	intergenic	-0.38
	chr10	43015248	intron/ORF	0.08

	chr10	43015315	intron/ORF	1.50*
	chr10	43016273	intron/ORF	-0.22
ZNF536	chr19	31029957	intron/ORF	-0.75*
	chr19	31048965	intron/ORF	0.28
	chr19	31204492	intergenic	0.04
**Group II transcriptionally regulated genes**				
ADAMTS1	chr21	28208188	intergenic	0.55
	chr21	28208606	3‘UTR	0.31*
	chr21	28208731	3‘UTR	-0.42*
	chr21	28209188	3‘UTR	-0.27*
	chr21	28209329	3‘UTR	-0.42*
	chr21	28209867	3‘UTR	0.28
ATF7IP2	chr16	10481596	intron/ORF	-0.61*
	chr16	10577494	3‘UTR	0.44*
BAIAP2	chr17	79083172	3‘UTR	0.23
	chr17	79084312	intron/ORF	0.23*
	chr17	79090024	3‘UTR	-0.46*
CBX6	chr22	39257467	3‘UTR	-0.11*
	chr22	39260350	3‘UTR	0.50*
CHST8	chr19	34115093	intron/ORF	0.41
	chr19	34115402	intron/ORF	1.54*
	chr19	34243616	intron/ORF	-0.60
	chr19	34264413	intergenic	-0.08
CREB5	chr7	28450376	intron/ORF	0.16*
	chr7	28451325	intron/ORF	0.25
	chr7	28453742	intron/ORF	0.06
	chr7	28859176	3‘UTR	-0.14
	chr7	28865505	3‘UTR	-0.55*
CD27-AS1	chr12	6548332	intron/ORF	0.64*
	chr12	6556287	intron/ORF	0.01
	chr12	6556793	intron/ORF	-0.53*
DLX2	chr2	172962927	intergenic	0.27*
	chr2	172963003	intergenic	0.13
	chr2	172964449	3‘UTR	-0.66*
EFNB2	chr13	107142078	intergenic	-0.03
	chr13	107142353	3‘UTR	-0.17
	chr13	107142546	3‘UTR	-0.14
	chr13	107142811	3‘UTR	-0.22
	chr13	107158394	intron/ORF	1.29*
	chr13	107182960	intron/ORF	0.90*
ELL2	chr5	95220802	3‘UTR	-0.16*
	chr5	95223088	3‘UTR	0.25*
	chr5	95223250	3‘UTR	-0.11
FAM111B	chr11	58891819	intron/ORF	-0.38
	chr11	58894883	intergenic	-0.03
	chr11	58894934	intergenic	-0.02
	chr11	58895851	intergenic	0.59*
FOSL2	chr2	28635799	3‘UTR	1.11
	chr2	28639557	3‘UTR	0.18

	chr2	28639757	3‘UTR	0.18
	chr2	28640177	3‘UTR	-0.33*
FOXO3	chr6	108960891	intron/ORF	0.01
	chr6	109001954	3‘UTR	-0.05
	chr6	109005976	3‘UTR	-0.28
	chr6	109070182	intergenic	1.28*
HIST1H2BK	chr6	27103705	intergenic	0.26
	chr6	27105074	intergenic	0.44*
	chr6	27106071	intergenic	-0.10*
ICAM5	chr19	10407452	3‘UTR	0.44*
	chr19	10446923	intergenic	-0.50*
ID4	chr6	19839642	3‘UTR	-0.54*
	chr6	19840564	3‘UTR	0.12
	chr6	19840914	3‘UTR	0.08
	chr6	19842424	intergenic	0.01
MAFA	chr8	144510234	3‘UTR	0.07
	chr8	144510533	3‘UTR	0.08
	chr8	144510650	3‘UTR	-0.69*
	chr8	144511018	3‘UTR	0.36
MCL1	chr1	150547035	3‘UTR	-0.10*
	chr1	150548437	3‘UTR	0.13*
	chr1	150548542	3‘UTR	-0.04
	chr1	150549663	3‘UTR	0.22
MSL1	chr17	38286547	intron/ORF	0.17*
	chr17	38293040	intergenic	-0.30*
NAT8L	chr4	2066807	3‘UTR	0.33
	chr4	2067635	3‘UTR	0.31*
	chr4	2070815	3‘UTR	-0.42*
USP53	chr4	120135275	intron/ORF	0.34*
	chr4	120215783	3‘UTR	-0.32*
	chr4	120215900	3‘UTR	-0.29
RP11-480I12.5	chr1	202822014	intron/ORF	0.22*
	chr1	202826574	3‘UTR	-0.34*
ZBTB47	chr3	42707643	3‘UTR	-0.57*
	chr3	42709072	intergenic	0.24*
ZC3HAV1	chr7	138728266	3‘UTR	-0.27*
	chr7	138745086	3‘UTR	0.28*
**Group III transcriptionally regulated, posttranscriptionally buffered genes**				
AGAP3	chr7	150820725	intron/ORF	-0.15*
	chr7	150841521	3‘UTR	0.13*
BMP7	chr20	55743807	3‘UTR	0.16*
	chr20	55745553	3‘UTR	-0.11*
CA8	chr8	61099905	3‘UTR	0.02
	chr8	61100664	3‘UTR	0.35
	chr8	61101531	3‘UTR	-0.42*
	chr8	61102101	3‘UTR	0.08
	chr8	61102217	3‘UTR	0.30
C10orf76	chr10	103605360	3‘UTR	0.11
	chr10	103606185	3‘UTR	0.07

	chr10	103789206	3‘UTR	-0.49*
C20orf96	chr20	251502	intergenic	0.13
	chr20	251559	3‘UTR	0.39
	chr20	270072	intron/ORF	-0.39*
EPHA8	chr1	22903531	intron/ORF	0.51
	chr1	22916110	3‘UTR	-0.17*
FAXC	chr6	99720796	intergenic	-0.48
	chr6	99728536	3‘UTR	0.08*
LHPP	chr10	126187083	intron/ORF	-0.44*
	chr10	126205942	intron/ORF	0.54
	chr10	126302709	3‘UTR	0.08
RAI1	chr17	17657994	intron/ORF	-0.67*
	chr17	17714763	3‘UTR	0.23*
RP11-159D12.5	chr17	56066398	3‘UTR	0.35*
	chr17	56078282	intron/ORF	-0.11
	chr17	56078436	intron/ORF	-0.06
	chr17	56080721	intron/ORF	-0.06
SLC25A37	chr8	23389437	intron/ORF	-0.03
	chr8	23425094	intron/ORF	-0.77*
	chr8	23429716	3‘UTR	0.21
	chr8	23430061	3‘UTR	-0.08
	chr8	23432974	3‘UTR	0.04
TCEA2	chr20	62695475	intron/ORF	-0.14*
	chr20	62695530	intron/ORF	0.04
	chr20	62703699	intron/ORF	0.11
TRAF2	chr9	139821059	intergenic	0.05*
	chr9	139829277	intergenic	-0.61
TRIM33	chr1	114935415	3‘UTR	0.15
	chr1	114938543	3‘UTR	0.32
	chr1	114940241	3‘UTR	-0.42
	chr1	114947448	intron/ORF	-1.08*

*$p_{adj} < 0.1$

Supplemental Table S6. 3'T-fill barcodes

primer name	sequence (5'→ 3')
P5_dT16VN	[Btn]AATGATACGGCGACCACCGAGATCTACACTCTTTCCCTACACGACGCTCTTCCGAT CTTTTTTTTTTTTTTTTTVN
P7_T1_for_Mpx_01	CAAGCAGAAGACGGCATACGAGATCGGTCTCGGCATTCCTGCTGAACCGCTCTTCCGA TCTCGTGAT *T
P7_T1_for_Mpx_02	CAAGCAGAAGACGGCATACGAGATCGGTCTCGGCATTCCTGCTGAACCGCTCTTCCGA TCTAAGCTA *T
P7_T1_for_Mpx_03	CAAGCAGAAGACGGCATACGAGATCGGTCTCGGCATTCCTGCTGAACCGCTCTTCCGA TCTGTAGCC *T
P7_T1_for_Mpx_04	CAAGCAGAAGACGGCATACGAGATCGGTCTCGGCATTCCTGCTGAACCGCTCTTCCGA TCTTACAAG *T
P7_T1_for_Mpx_05	CAAGCAGAAGACGGCATACGAGATCGGTCTCGGCATTCCTGCTGAACCGCTCTTCCGA TCTACATCG *T
P7_T1_for_Mpx_06	CAAGCAGAAGACGGCATACGAGATCGGTCTCGGCATTCCTGCTGAACCGCTCTTCCGA TCTGCCTAA *T
P7_T1_rev_Mpx_01	[Phos]ATCACGAGATCGGAAGAGCGGTTCAGCAGGAATGCCGAGACCGATCTCGTATGCC GTCTTCTG CTTG
P7_T1_rev_Mpx_02	[Phos]TAGCTTAGATCGGAAGAGCGGTTCAGCAGGAATGCCGAGACCGATCTCGTATGCC GTCTTCTG CTTG
P7_T1_rev_Mpx_03	[Phos]GGCTACAGATCGGAAGAGCGGTTCAGCAGGAATGCCGAGACCGATCTCGTATGCC GTCTTCTG CTTG
P7_T1_rev_Mpx_04	[Phos]CTTGTAAGATCGGAAGAGCGGTTCAGCAGGAATGCCGAGACCGATCTCGTATGCC GTCTTCTG CTTG
P7_T1_rev_Mpx_05	[Phos]CGATGTAGATCGGAAGAGCGGTTCAGCAGGAATGCCGAGACCGATCTCGTATGCC GTCTTCTG CTTG
P7_T1_rev_Mpx_06	[Phos]TTAGGCAGATCGGAAGAGCGGTTCAGCAGGAATGCCGAGACCGATCTCGTATGCC GTCTTCTG CTTG

[Btn] biotinylated nucleotide, [Phos] 5' phosphate, *s-linkage between bases, barcodes are underlined

Supplementary Table S7. Sequences of q-RT-PCR primers

**GAPDH**	**fwd:** 5'-TGAGCTTGACAAAGTGGTCG-3'
	**rev:** 5'-GGCTCTCCAGAACATCATCC-3'
**ZNF79**	**fwd:** 5'-GACAGGTTCAAGGAGGGGAT-3'
	**rev:** 5'-CGCCTTCCCTTTCTTCCAAC-3'
**ZNF557**	**fwd:** 5'-AAACCTAGGCTGATCTCCCA-3'
	**rev:** 5'-TGCAGCTTAGGAGTTAACCCT-3'
**ZNF670**	**fwd:** 5'-TCCTGGGAGAAATCTAAGCAGT-3'
	**rev:** 5'-GTTTCTCCATATTGACTGCCTTC-3'
**RBM5**	**fwd:** 5'-GTGATGATCGGAGGGGTGAT-3'
	**rev:** 5'-TCTTCGGATCGGTCACTGTT-3'
**GADD45B**	**fwd:** 5'-GAGTCGGCCAAGTTGATGAA-3'
	**rev:** 5'-ATCCTCCTCCTCCTCGTCAA-3'
**FOS**	**fwd:** 5'-TTACTACCACTCACCCGCAG-3'
	**rev:** 5'-AGTGACCGTGGGAATGAAGT-3'
**EGR1**	**fwd:** 5'-AGCAGCACCTTCAACCCTC-3'
	**rev:** 5'-CCAGCACCTTCTCGTTGTTC-3'
**PLIN2**	**fwd:** 5'-ATGAGTCCCACTGTGCTGAG-3'
	**rev:** 5'-GTCTGGAGCTGCTGAGTCA-3'
**ACTG1**	**fwd:** 5'-ATGAGTCCCACTGTGCTGAG-3'
	**rev:** 5'-GTCTGGAGCTGCTGAGTCA-3'

**CBFB**	**fwd:** 5’-GCCCATCTTTACATACACA-3’
	**rev:** 5’-ACTTCAAATTATTACTGGCTAC-3’
**HPRT**	**fwd:** 5’-GACCAGTCAACAGGGGACAT-3’
	**rev:** 5’-AACACTTCGTGGGGTCCTTTTC-3’
**RORA short + long 3’UTR**	**fwd:** 5’-ATCGGTGACTGGTGTGCATA-3’
	**rev:** 5’-GGACCCTTTTCATGCCATCC-3’
**RORA long 3’UTR**	**fwd:** 5’-TCCTTAACTGCCTCTCTTGGA-3’
	**rev:** 5’-ATGTGCTAGCCATTGTGCAG-3’
**SETD4 long**	**fwd:** 5’-ACTCACAGCCCTTAAGTTGTT -3’
	**rev:** 5’-TGGGCTATGTCCAAACTTGTC-3’
**SETD4 short**	**fwd:** 5’-GCGGGATTGAGCATTTCCAC-3’
	**rev:** 5’-AGAACTTCCGCAGAGTTTTCG-3’
**SNX5 long**	**fwd:** 5’-TAAGGCCCGGTTAAAGAGCA-3’
	**rev:** 5’-CTGCCACTCTCTTCCGTTTG-3’
**SNX5 short**	**fwd:** 5’-CAGCAAGAACCAGAGACGC-3’
	**rev:** 5’-AAGAACATTCCGGGGTCTCC-3’
**TRMT44 short**	**fwd:** 5’-TCCACATCCTGAGCAGTGAG-3’
	**rev:** 5’- TGAGATCAAGGGCATGCGTA-3’
**TRMT44 long**	**fwd:** 5’-CAGAAGTAGCCAACGAGCTG-3’
	**rev:** 5’-GCGGATGTGAACTCTCCCAT-3’
**TROAP short**	**fwd:** 5’-GTACGCTCTCAGAAACGCAC-3’
	**rev:** 5’-GGCGTTGAATATTGAGCGGT-3’

**TROAP long**	**fwd:** 5’-GGACGATGAGTGTGCCTTTT-3’
	**rev:** 5’-CTGGAATGAAACACAGGGCA-3’
**MARK1 long**	**fwd:** 5’-TCACCGTGATCTTAAGGCTGA-3’
	**rev:** 5’-TGTCCAATTTGTTCCCAACTGT-3’
**MARK1 extended exon**	**fwd:** 5’-ACTCAGCTAAATCCTACCAGTCT-3’
	**rev:** 5’-TCAAGCAACTACTTGGTAAGTCA-3’
**HP1BP3 long**	**fwd:** 5’-CCATGAATGAGCCGAAGACC-3’
	**rev:** 5’-CATCCACCCATTCTTTTCGCA-3’
**HP1BP3 extended exon**	**fwd:** 5’-ACTCGCCTTTGTGAACCTAA-3’
	**rev:** 5’-AGCACTTCTCCACAACCAGG-3’
**ILF3 long**	**fwd:** 5’-ACTGGGATGAAGTTGGAGGG-3’
	**rev:** 5’-GGGGCCTCCTTTCATCCTAG-3’
**ILF3 short**	**fwd:** 5’-GGCTACATGAATGCCGGTG-3’
	**rev:** 5’- AAGTCACTGTAGCCTGCTGT-3’

pre-mRNA:

**preGAPDH**	**fwd:** 5’-AGGGCCCTGACAACTCTTTT-3‘
	**rev:** 5’-AGGGGTCTACATGGCAACTG-3‘
**preZNF79**	**fwd:** 5’-CCACCAAGGGAACACAACAG-3’
	**rev:** 5’-GGGCATCTCTACACATGGGT-3’
**preZNF557**	**fwd:** 5’-TCCTCTGACAGCATTCAATGT-3‘
	**rev:** 5‘-TGCCGTGTAAGGGAAGATTT-3‘

**preZNF670**	**fwd:** 5'-ACAGAGATGTGATGCAAGAAATC-3'
	**rev:** 5'-CAAAACACTTGCGTTGTAATCAA-3'
**preRBM5**	**fwd:** 5'-CTGTGGGCCGTGTGTATTTT-3'
	**rev:** 5'-CATCACGGTCATCCCTGTCT-3'
**preRORA**	**fwd:** 5'-AGGTGATGTGGCAATTGTGT-3'
	**rev:** 5'-CGACACCTTCCTTACCAAGC-3'
**preGADD45B**	**fwd:** 5'-TGACCCATCCCTACCCTTTG-3'
	**rev:** 5'-CTCCTCCTCCTCGTCAATGG-3'
**preFOS**	**fwd:** 5'-TTACTACCACTCACCCGCAG-3'
	**rev:** 5'-TGTCTCCTCCTCCGCGAC-3'
**preEGR1**	**fwd:** 5'-AGCAGCACCTTCAACCCTC-3'
	**rev:** 5'-GATGGTGGCGAAAGGGTTC-3'
**preACTG1**	**fwd:** 5'-CTGGCTGTCTTTGCAGATCA-3'
	**rev:** 5'-TCAATTAACCCATGCAGCAA-3'
**preCBFB**	**fwd:** 5'-TTTTCATCCTTTTCTGTGTCTTA-3'
	**rev:** 5'-CCATCCAGTCTTTGGAGATCA-3'
**preHPRT**	**fwd:** 5'-ACGTCAGTCTTCTCTTTTGTAAT-3'
	**rev:** 5'-ACACTTCGTGGGGTCCTTTT-3'
**prePLIN2**	**fwd:** 5'-ACCTCTCATGGGTAGAGTGG-3'
	**rev:** 5'-CCAACAAATGACCGTCCCAG-3'
**preSETD4**	**fwd:** 5'-ACTCACAGCCCTTAAGTTGTT-3'
	**rev:** 5'-ACATTCGCTACATGGGCAAG-3'

**preSNX5**	**fwd:** 5'- TAAGGCCCGGTTAAAGAGCA -3'
	**rev:** 5'-TAAGGCAGAGGGGTGAAGTG-3'
**preTRMT44**	**fwd:** 5'-CAGAAGTAGCCAACGAGCTG-3'
	**rev:** 5'-AACTTGCTGCTTCTGTCAGA-3'
**preTROAP**	**fwd:** 5'-GGACGATGAGTGTGCCTTTT-3'
	**rev:** 5'-ACCTCTGTGAAGAAGCCC-3'
