# Supplementary material for: The differential expression of alternatively polyadenylated transcripts is a common stress-induced response mechanism that modulates mammalian mRNA expression in a quantitative and qualitative fashion
Source: RNA. 2016 Sep;22(9):1441–53. doi: 10.1261/rna.055657.115 (PMC4986898; doi:10.1261/rna.055657.115)
Supplement: Supplemental Material [file supp_055657.115_SuppFigs.pdf]

Supplemental Figure S1

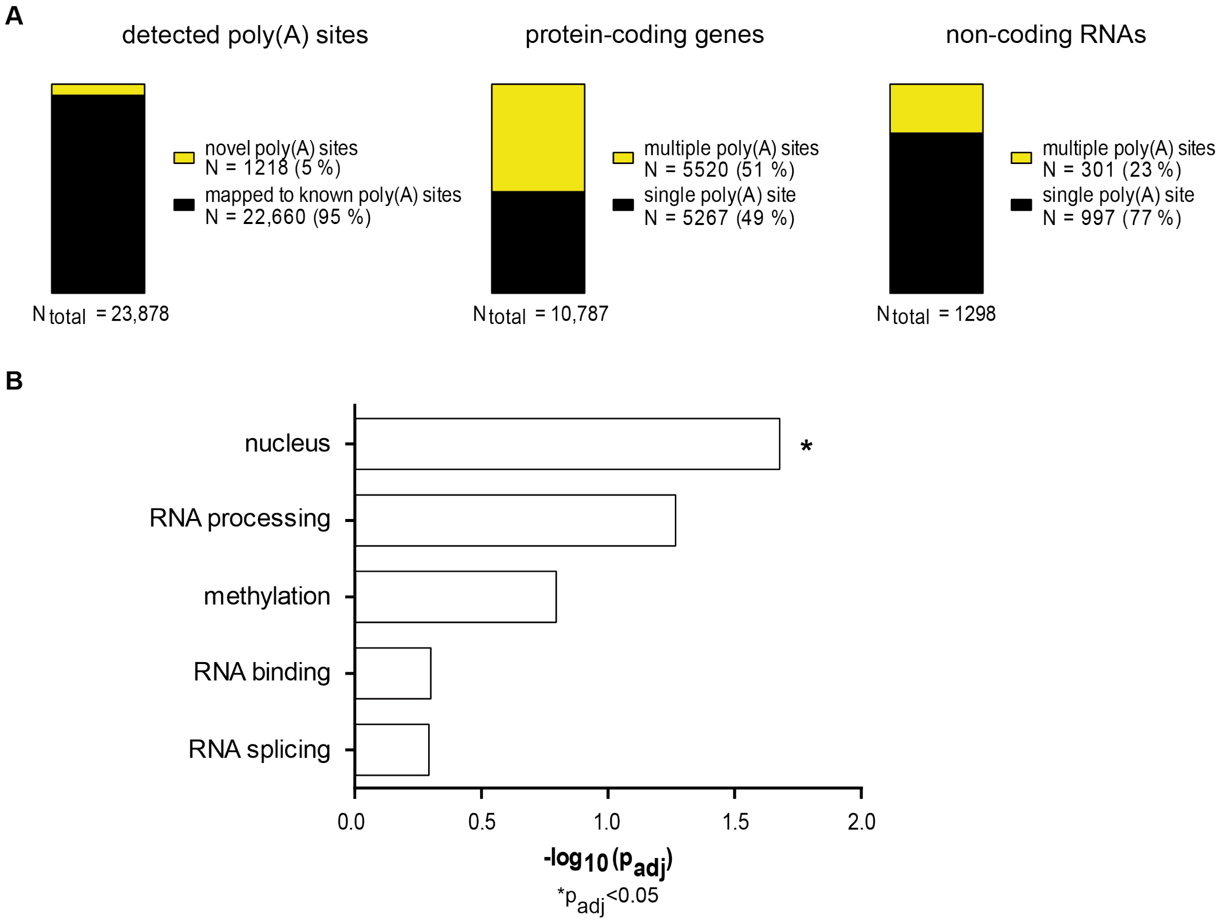

Supplemental Figure S1.

A 3’T-fill analysis detected high-confidence functional poly(A) sites in protein-coding genes and non-coding RNAs and shows that 51% of protein-coding genes and 23% of non-coding RNAs are polyadenylated at multiple poly(A) sites.

B Clustering of genes undergoing stress-induced APA according to biological function, cellular compartment and molecular mechanism using the DAVID software.

Supplemental Figure S2

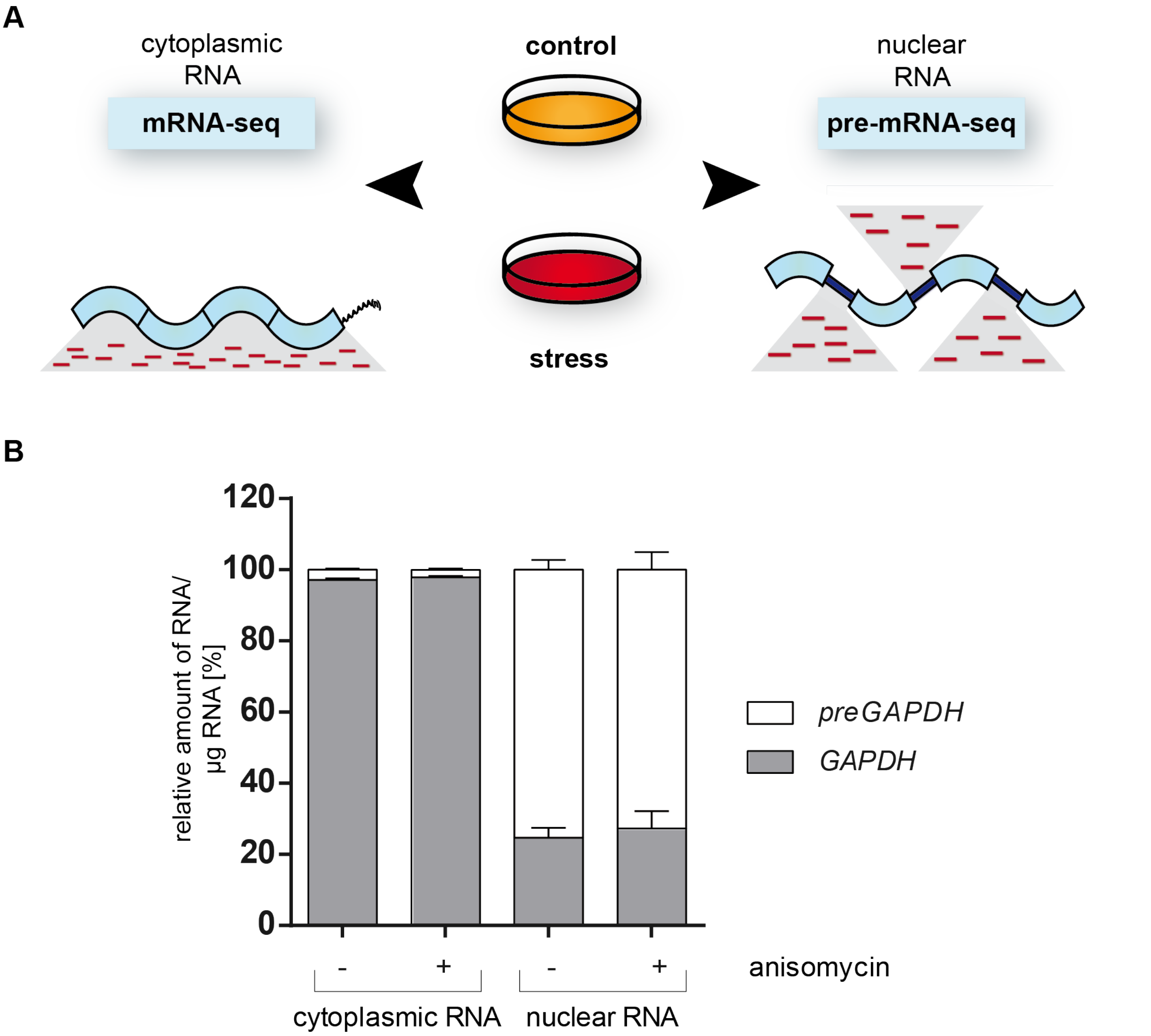

Supplemental Figure S2.

A mRNA-seq analysis of cytoplasmic RNA and pre-mRNA-seq analysis of nuclear RNA quantified stress-induced changes in mRNA and pre-mRNA abundance. In mRNA-seq, all mapped sequencing reads (*red lines*) were considered for downstream analyses, while only mapped reads derived from introns were kept for further analyses in pre-mRNA-seq.

B q-RT-PCRs measuring relative *preGAPDH* and *GAPDH* abundances in nuclear and cytoplasmic RNA samples to control the quality of the fractionation to control for the quality of the cytoplasmic RNA- and the nuclear pre-RNA samples.

Supplemental Figure S3

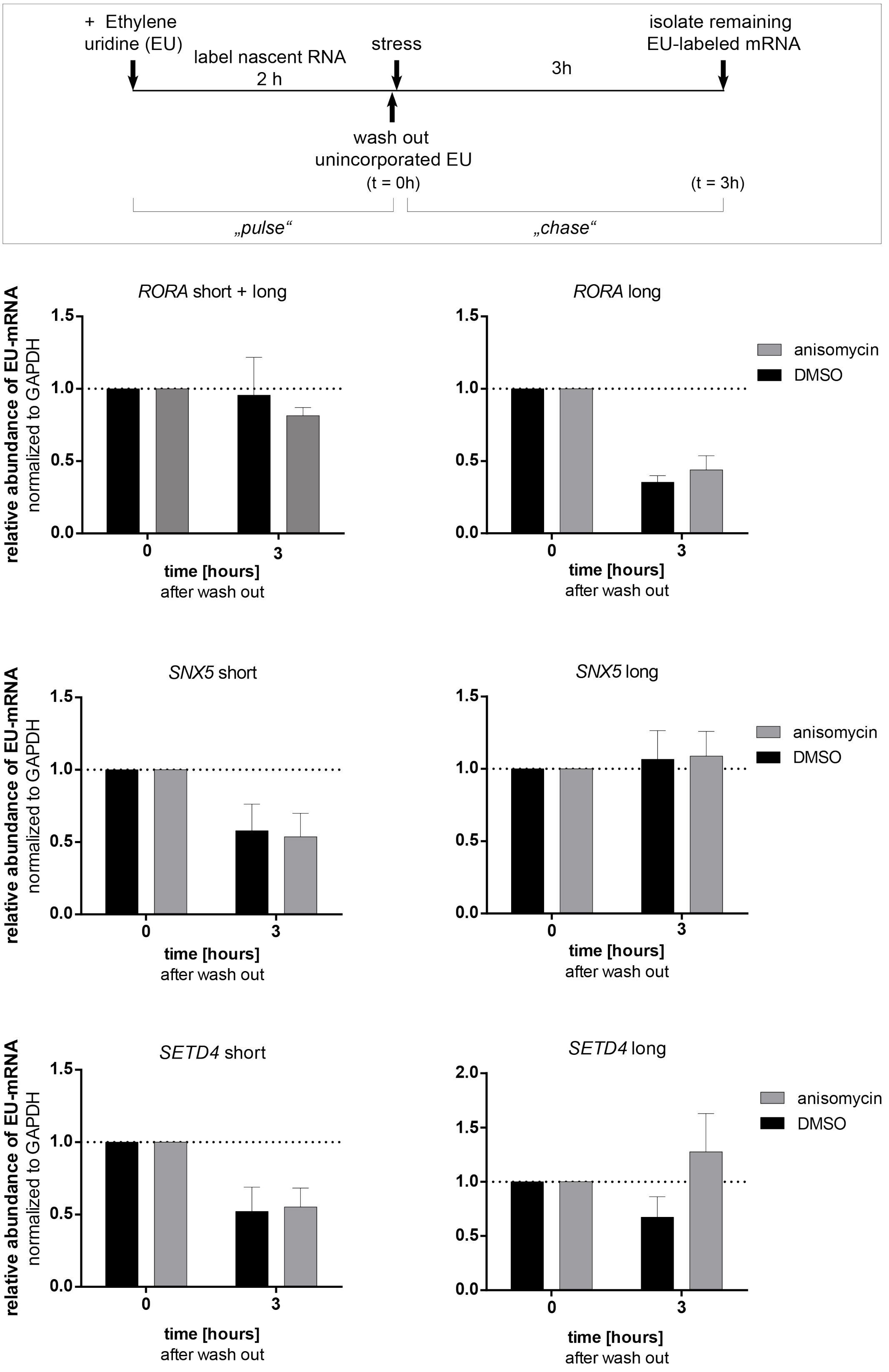

Supplemental Figure S3.

Assays measuring the stability of the short and the long isoforms of *RORA*, *SNX5* and *SETD4* mRNAs after 3 hours of DMSO or anisomycin treatment. Nascent RNA was labeled with the nucleotide analogue ethylene uridine (EU) ("pulse"), the label was washed out and cells were subsequently treated with DMSO or anisomycin. EU-labeled RNA was isolated before (t = 0h after wash out) and after treatment (t = 3h after wash out) ("chase") and EU-mRNA levels were assessed in qRT-PCRs using primers specific for the long or the short mRNA isoforms.

Supplemental Figure S4

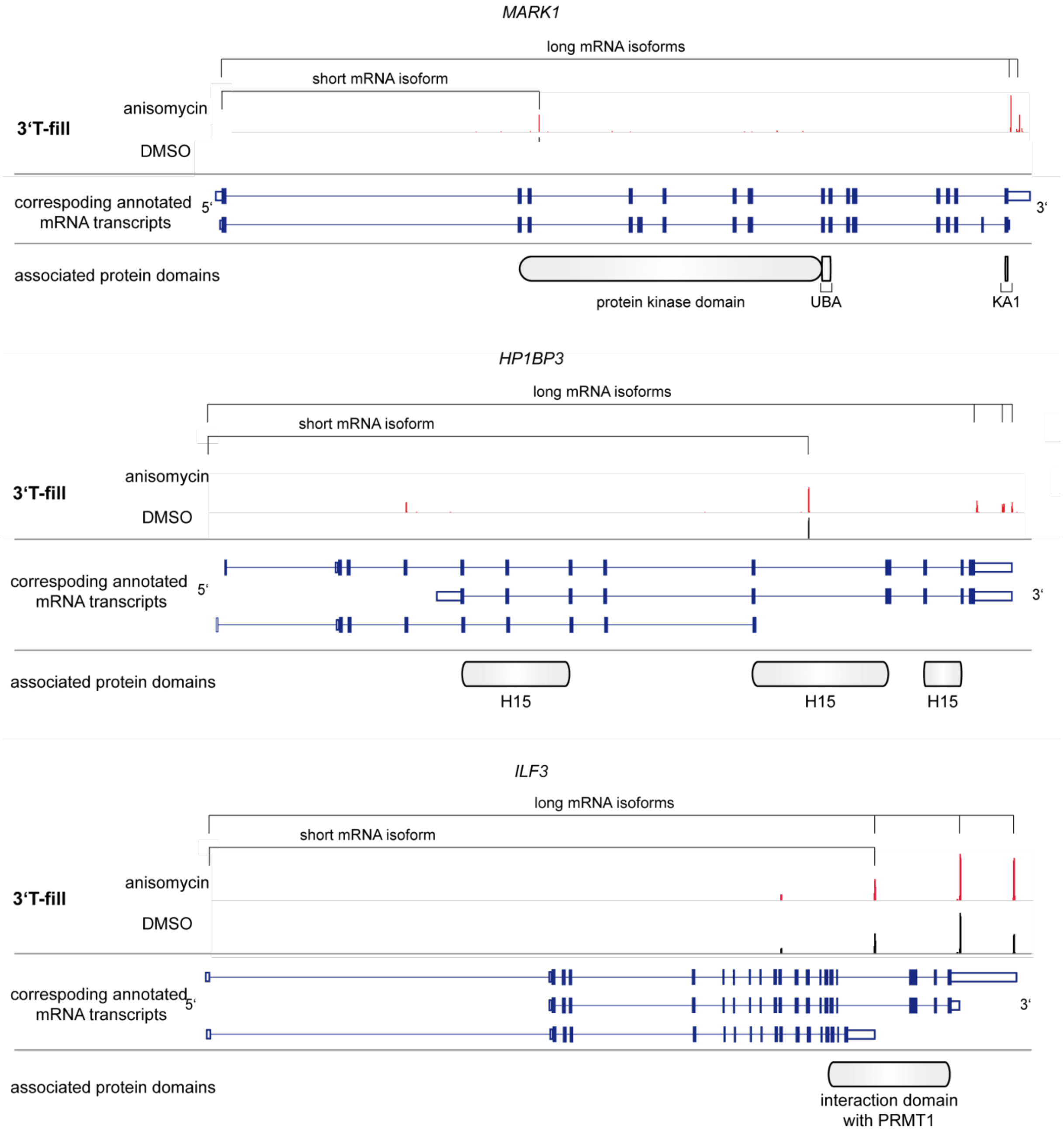

Supplemental Figure S4. 3’T-fill data of the *MARK1*, *HP1BP3* and *ILF3* genes in anisomycin- (*red lane*) and DMSO-treated control (*black lane*) cells and the respective stress-regulated short and long isoforms. Schematic representation of the affected *MARK1*, *HP1BP3* and *ILF3* transcripts as annotated in Ensembl and the associated encoded functional protein domains (below).

Supplemental Figure S5

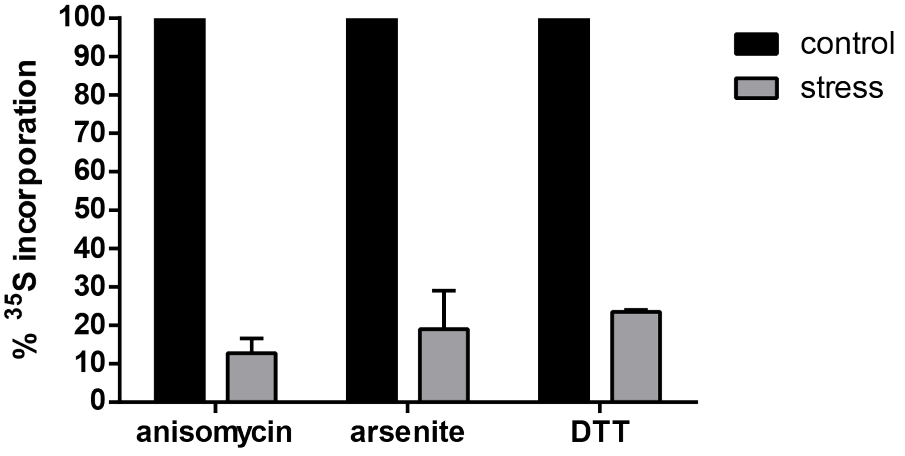

Supplemental Figure S5. ^{35}S-methionine assay measuring *de novo* protein synthesis after DTT, arsenite and anisomycin treatment, respectively (data points represent mean + SEM, n = 3). ^{35}S-methionine incorporation was normalized to DMSO-treated (anisomycin) or untreated control cells (arsenite, DTT).
